# Integrative *In Silico* and Experimental Evaluation of *Borassus flabellifer* Immature Endosperm for Dual Modulation of Diabetes and Hypothyroidism

**DOI:** 10.3390/nu18121931

**Published:** 2026-06-15

**Authors:** Shaikh Shahinur Rahman, Md. Rakibul Hasan Rahat, Anuwatchakij Klamrak, Md. Rasul Karim, Muzahid Fahim, Md. Imtiajul Haque, Arafat Bin Muhammad, Sinthia Doly Shurmi, Akbor Hossain, Joy Baisnab, Shakh M. A. Rouf, Yutthakan Saengkun, Jureerut Daduang, Sakda Daduang

**Affiliations:** 1 Division of Pharmacognosy and Toxicology, Faculty of Pharmaceutical Sciences, Khon Kaen University, Khon Kaen 40002, Thailand; shahin@anft.iu.ac.bd (S.S.R.); anuwat_kla@yahoo.com (A.K.); yutthakan_s@kkumail.com (Y.S.); 2 Department of Applied Nutrition and Food Technology, Faculty of Biological Sciences, Islamic University, Kushtia 7003, Bangladesh; rakibulrahat5@gmail.com (M.R.H.R.); muzahidfahim737@gmail.com (M.F.); imtiazsakib531@gmail.com (M.I.H.); arafat.anft24.iu.bd@gmail.com (A.B.M.); sinthiashurmi@gmail.com (S.D.S.); akborhossain.anft@std.iu.ac.bd (A.H.); abdurrouf@anft.iu.ac.bd (S.M.A.R.); 3 Department of Pharmacy, Islamic University, Kushtia 7003, Bangladesh; mrk.kamol@gmail.com (M.R.K.); joybaisnab.pharm.iu@gmail.com (J.B.); 4 Department of Clinical Chemistry, Faculty of Associated Medical Sciences, Khon Kaen University, Khon Kaen 40002, Thailand; jurpoo@kku.ac.th; 5 Protein and Proteomics Research Center for Commercial and Industrial Purposes (ProCCI), Khon Kaen University, Khon Kaen 40002, Thailand

**Keywords:** *Borassus flabellifer*, GC-MS, phytochemicals, molecular docking, TRβ1, PPARγ, AMPK, diabetes mellitus, hypothyroidism, metabolic disorders, functional food

## Abstract

**Background/Objectives**: The present study estimated the potential therapeutic effects of *Borassus flabellifer* immature endosperm extract (BFE) on the metabolic disorders of diabetes and hypothyroidism using a mixed research design. **Methods**: Characterization of phytochemicals via GC-MS demonstrated a highly abundant list of bioactive compounds, and it encompassed phenolic derivatives, methylxanthines, fatty acids, and inositol-related compounds. Molecular docking indicated that the major phytoconstituents showed positive binding affinities to the most vital metabolism and endocrine receptors, namely, TRβ1, PPARγ, and AMP-activated protein kinase (AMPK). Notably, both compounds C1 and C2 were highly affined towards TRβ1 (−7.8 and −7.6 kcal/mol), which is attributed to interactions in the active site through hydrogen bonding and hydrophobic responses, which means that the identified compounds were found to have good predicted interactions with some metabolic- and thyroid-associated targets and could be used to form preliminary hypotheses for further mechanistic studies. The in vivo data showed that the disease-induced groups were marked by hyperglycemia, imbalance in thyroid hormones, and dyslipidemia, as well as liver, kidney, and heart dysfunction. BFE caused significant decreases in these changes, which were also observed through improvements in fasting blood glucose, T3, T4, and TSH; partial restoration of lipid profiles; and dampening of liver and kidney injury signalers. The cardiac risk indices were also reduced significantly after BFE administration. Positive changes in body weight gain, feed ratio, and metabolic ratio further reflected better physiological stability. **Results**: These findings were corroborated by histopathological analysis, which showed that the tissue architecture of the pancreas, liver, kidney, and heart had significantly recovered in the study. BFE still showed constant therapeutic activity even though the magnitude of response was attenuated when combined disease conditions were used. **Conclusions**: Comprehensively, the results indicate that BFE potentially plays a role in the amelioration of metabolic and endocrine abnormalities of diabetic and hypothyroid conditions. These observations should be regarded as hypothesis-generating, as further mechanistic and translational studies are needed to substantiate their biological relevance.

## 1. Introduction

Diabetes mellitus and thyroid dysfunction are among the most common endocrine disorders worldwide, and their coexistence presents a complex clinical challenge. Type 2 diabetes mellitus (T2DM) is characterized by chronic hyperglycemia, insulin resistance, and progressive β-cell dysfunction, while hypothyroidism is associated with reduced metabolic rate, dyslipidemia, and impaired glucose metabolism. Increasing clinical and experimental evidence indicates a strong physiological interplay between these two conditions, where thyroid hormones influence insulin sensitivity, glucose utilization, and lipid metabolism, thereby exacerbating metabolic dysregulation when both disorders coexist [1,2]. This interrelationship highlights the need for therapeutic strategies capable of targeting both glycemic control and thyroid function simultaneously.

Current pharmacological interventions for diabetes and hypothyroidism are effective but often require long-term administration and may be associated with adverse effects, limited accessibility, and reduced patient compliance, particularly in resource-limited settings. In this context, there is growing interest in plant-derived bioactive compounds and functional nutraceuticals as alternative or complementary therapeutic options. These natural compounds often exhibit multi-target actions, including antioxidant, anti-inflammatory, antihyperglycemic, and lipid-lowering effects, which are beneficial in managing complex metabolic disorders [3,4].

Among underutilized plant resources, *Borassus flabellifer* (palmyra palm) has attracted attention due to its nutritional richness and traditional medicinal use. *B. flabellifer* immature endosperm is particularly notable for its content of carbohydrates, phenolic compounds, and bioactive metabolites [5,6]. Emerging evidence suggests that different parts of this plant possess antidiabetic, antihyperlipidemic, and antioxidant activities, supporting its role in metabolic regulation [7,8,9]. However, systematic investigations focusing specifically on the endosperm and its mechanistic effects on combined metabolic–endocrine disorders remain limited.

In the present study, GC–MS analysis of the endosperm extract revealed a diverse array of phytochemical constituents, including compounds known for their metabolic regulatory potential. The presence of such bioactive molecules suggests the possibility of synergistic interactions contributing to the observed therapeutic effects. This aligns with recent perspectives that emphasize the importance of whole-extract functionality rather than isolated compounds in achieving effective metabolic modulation [10].

Mechanistically, metabolic homeostasis is regulated by several key signaling pathways, including AMP-activated protein kinase (AMPK), peroxisome proliferator-activated receptors (PPARs), and insulin signaling cascades. Activation of AMPK enhances glucose uptake and fatty acid oxidation, while PPARγ plays a critical role in lipid metabolism and insulin sensitivity [11,12]. The pathways are dysregulated, which is a central process in the formation of diabetes and thyroid-related metabolic disturbances [13,14]. Additionally, thyroid hormone receptors (TRs), particularly TRβ1, are essential regulators of metabolic rate and lipid homeostasis, further linking thyroid function with systemic metabolism [15].

Beyond molecular signaling, recent research has underscored the role of oxidative stress and inflammation as common pathological mediators in both diabetes and hypothyroidism. Elevated reactive oxygen species (ROS) and pro-inflammatory cytokines contribute to insulin resistance, β-cell dysfunction, and altered thyroid hormone synthesis. Natural antioxidants derived from plant sources have shown promise in mitigating these effects, thereby improving metabolic outcomes [16,17,18].

Importantly, advances in in silico approaches, such as molecular docking and network pharmacology, have provided powerful tools for predicting interactions between bioactive compounds and target proteins. These methods allow for the identification of potential molecular targets, including enzymes and receptors involved in glucose and thyroid hormone regulation, thereby offering mechanistic insights that complement experimental findings [19,20]. When integrated with in vivo and in vitro validation, such approaches can significantly enhance the understanding of functional nutraceuticals.

Despite these advancements, there remains a notable gap in the literature regarding integrative studies that combine computational prediction with experimental validation to investigate the dual modulation of diabetes and hypothyroidism. Most studies focus on either antidiabetic or thyroid-related effects independently, with limited exploration of their combined regulation using a single natural source. Furthermore, the mechanistic basis of action of many traditional plant-based remedies remains insufficiently characterized.

In this context, the present study aimed to conduct an integrative in silico analysis and to examine the effect of BFE on interrelated metabolic and endocrine abnormalities in diabetes, hypothyroidism and their associated condition. Hence, several biochemical, hormonal and histopathological parameters were assessed to represent an integrated approach for evaluating its biological effects on glycemic management, thyroid function, lipid metabolism and organ integrity.

## 2. Materials and Methods

### 2.1. Plant Collection and Preparation of Extract

Fresh *B. flabellifer* fruit endosperm was collected from local areas of Kushtia District, located between 23°42′ and 24°12′ north latitudes and between 88°42′ and 89°22′ east longitudes in Bangladesh. Soft seed coats were removed, and immature endosperms were collected and dried in a laboratory oven at 50 °C for 48–72 h and then ground into a fine powder using an electric laboratory blender and preserved for experimental use.

### 2.2. Extraction Procedure

Twenty grams of powdered plant material was placed in a 500 mL Erlenmeyer flask and extracted with 200 mL of 30% (*v*/*v*) hydroethanolic solution. The mixture was shaken continuously at 100 rpm for 48 h at room temperature (25 °C) using an incubator shaker. After extraction, the mixture was centrifuged at 7500× *g* for 20 min at room temperature to separate the supernatant. The clear extract was rotary-evaporated to a partial concentrate to remove ethanol. The aqueous extract was freeze-dried at −110 °C for 48 h to obtain a dry extract, which was stored at −80 °C until analysis. The extraction conditions were determined based on previous work reporting that a 30% hydroethanolic system is considered a more effective system for the extraction of polar and semipolar bioactive compounds, such as phenolics, flavonoids, sugars and inositol derivatives, from *Borassus flabellifer* and its related plant materials [21].

### 2.3. Gas Chromatography–Mass Spectrometry (GC-MS) Analysis

The analysis via GC-MS was performed using a combined 8890 Gas Chromatography System (Agilent 19091S-433UI:2489267H, Agilent Technologies, Santa Clara, CA, USA) linked to an Agilent 7010B GC/TQ MS system, utilizing an HP-5MS UI fused silica column (5% phenyl methyl siloxane, 30 m × 250 μm × 0.25 μm). Helium served as the carrier gas at a flow rate of 1.72 mL/min. The oven temperature was set to 120 °C (with a hold of 2.00 min) and subsequently increased at a rate of 10 °C/min to 320 °C (with a hold of 5.00 min). The temperature of the ion source was 280 °C, the injection volume was 1.0 μL (with a hold time of 5.00 min), the injector temperature was 250 °C, and the split ratio was set to 20:1. A research project using ionization mass spectrometry was conducted at 70 eV. Mass spectra were recorded from 30 *m*/*z* to 550 *m*/*z* over 24 min. The entire run time was 27.0 min, including a solvent cut time of 3.0 min. The phytochemicals in the samples were recognized by comparing their retention time (min), peak area, peak height, and mass fragmentation patterns with the databases of genuine compounds kept in the National Institute of Standards and Technology (NIST) library 2020 [22,23].

### 2.4. Animals

Male Wistar rats were bought from the Animal House of the Faculty of Biological Sciences, Islamic University, Bangladesh, and weighed between 80 and 90 g. Before the start of the experimental procedure, the animals were kept for one week under controlled laboratory conditions. During this period, the rats were kept in polypropylene cages. They were given ad libitum access to standard laboratory food and clean water. During the study and in the period of acclimatization, the temperature was maintained at 24 ± 2 °C, and the relative humidity was at 45 ± 5%. A 12 h day/night cycle was also maintained.

### 2.5. Animal Diet

All the nutritionally balanced standardized diets were given to the experimental groups as per the recommendations of Rahman et al. (2021) [24]. This diet included 30% wheat flour, 10% fishmeal as a protein source, 20% rice polish, 10% oilseed cake, 21% wheat bran, 5% molasses, 0.5% vitamins, 1.5% common salt, and 2% soybean oil, which is regarded as a standard laboratory diet.

### 2.6. Experimental Design, Randomization, Blinding, and Animal Welfare

This study aims to assess the validity of the *B. flabellifer* immature endosperm (BFE) as a potential therapy for the dual condition of hyperglycemia and hypothyroidism. For this study, male Wistar rats (n = 8 per group) were equally and randomly divided into 10 experimental groups.
Group 1 (HC):Healthy control rats with no other treatment.Group 2 (HTC):Hypothyroid control rats; these were triggered by the introduction of 0.05% 6-propyl-2-thiouracil (PTU) for 4 weeks in the drinking water.Group 3 (DC):Diabetic control rats; these were triggered by an intraperitoneal single shot of 65 mg STZ (streptozotocin) per kg body weight.Group 4 (DHC): No treatment for combined diabetic and hypothyroid control rats.Group 5 (HBFE):Healthy rats with BFE (500 mg/kg/day, oral) treatment.Group 6 (HTBFE):Hypothyroid rats managed with BFE (500 mg/kg/day, orally).Group 7 (HTD):Hypothyroid rats with L-thyroxine (50 µg/kg/day, orally).Group 8 (DBFE):Diabetic rats managed with BFE (500 mg/kg/day, orally).Group 9 (DD):Diabetic rats with glibenclamide (5 mg/kg/day, orally).Group 10 (DHBFE):Diabetic–hypothyroid rats managed with BFE (500 mg/kg/day, orally).

A computer-generated randomization procedure performed by an investigator who was not involved in the treatment was used to randomly assign animals to the experimental groups. All animals were housed under standard room temperature (24 ± 2 °C), relative humidity (45% ± 5) and a 12 h light/dark cycle during the course of the experiment. To avoid site-specific environmental bias, cage locations were changed every so often within the animal facility.

To minimize circadian variation as well as procedural variance, drug administration and sample collection and biochemical measurements were conducted at the same time of day. All investigators involved in biochemical analyses, histopathological evaluation, microscopic scoring, and statistical analysis were blinded to the treatment allocation, while the personnel responsible for treatment administration knew the group allocation because of practical dosing needs. The observer and analytical bias were minimized by coding tissue sections and biological samples prior to analysis.

There was no exclusion of animals or experimental units or data points from the final statistical analysis. There were no unexpected mortalities or protocol deviations in all experimental animals that completed the study protocol. Rats were observed twice daily during the study for pain and/or distress, such as dehydration, lethargy, decreased ambulation, poor grooming, difficulty breathing, and inappetence or reduced water consumption. The humane endpoints were severe weakness, inability to take food and water, loss of body weight over 20%, severe respiratory issues, and suffering for an extended period that led to euthanasia. During the experimental period, none of the animals experienced the criteria of humane endpoints.

Fasting blood glucose (FBG) was used as the main outcome measure for the sample size selection and was the main metabolic outcome endpoint for the study. The sample size was determined based on previously published experimental studies with similar models of metabolic and endocrine dysfunction and was based on the Reduction principle of the 3Rs (i.e., minimizing animal numbers). All animal experiments were performed in compliance with the ARRIVE 2.0 guidelines and the institutional ethical guidelines on the care and use of laboratory animals.

BFE powder was suspended in 1 mL of distilled water and administered once daily via oral gavage to Groups 5, 6, 8, and 10. The groups without treatment (Groups 1–4) were given distilled water in the same amounts. Standard drugs were administered orally to Group 7 (L-thyroxine, 50 µg/kg/day) and Group 9 (glibenclamide, 5 mg/kg/day) as reference treatments.

The experimental period was 28 consecutive days. The animals were kept under the same standardized laboratory conditions for the entire duration of the study.

### 2.7. Induction of Diabetes and Hypothyroidism

Hypothyroidism was induced using a validated protocol involving the administration of 0.05% 6-propyl-2-thiouracil (PTU) in the drinking water for 28 consecutive days [25]. Successful induction was confirmed through serum hormone profiling. Rats were considered hypothyroid when their thyroid-stimulating hormone (TSH) levels exceeded 4.5 mU/L [26] and their free thyroxine (FT4) and free triiodothyronine (FT3) concentrations dropped below 19 pmol/L and 3.20 pmol/L, respectively [27].

Experimental diabetes was induced in overnight-fasted rats by a single intraperitoneal injection of streptozotocin (STZ) at a dose of 65 mg/kg body weight. Szkudelski [28] states that STZ should be fresh before injection and should be prepared in 0.1 M cold sodium citrate buffer (pH 4.5) to ensure its stability. Using a glucometer, the fasting blood glucose (FBG) levels were recorded 72 h post-injection. The rats were included in the experiment if they were diabetic (FBG levels > 250 mg/dL, >13.8 mmol/L, which indicates the destruction of the pancreatic β-cells). All control and experimental animals were housed separately in metabolic cages under standardized laboratory conditions to avoid cross-contamination and ensure accurate monitoring of food and water intake.

### 2.8. Biochemical and Hormonal Analysis

Weekly fasting blood samples (10–12 h fast) were collected from the tail vein. Blood glucose was measured using an Accu-chek Advantage^®^ glucometer (Roche Diagnosis, Mannheim, Germany) with glucose-oxidase/peroxidase strips. Upon completion of treatment, terminal blood was drawn from the retro-orbital plexus after overnight fasting. Serum was separated by centrifugation (300 rpm, 10 min) and stored at −80 °C until analysis.

Serum T3, T4, and TSH were quantified using rat-specific ELISA kits (Cusabio Biotech Co., Ltd., Wuhan, China). The reported intra-assay and inter-assay precision were 15%. T3 and T4 were measured via competitive inhibition ELISA, while TSH was quantified by sandwich ELISA, following manufacturer protocols.

Serum biochemical markers, including total cholesterol, triglycerides, high-density lipoprotein (HDL), and low-density lipoprotein (LDL), were evaluated using enzymatic colorimetric methods. Liver function enzymes—serum glutamic oxaloacetic transaminase (SGOT), serum glutamic pyruvic transaminase (SGPT), and alkaline phosphatase (ALP)—as well as a renal function marker (serum creatinine) and cardiac biomarkers (creatine kinase [CK] and creatine kinase-MB [CK-MB]) were measured using an automated biochemistry analyzer (BIOELAB AS-280; Dhaka, Bangladesh).

### 2.9. Histopathological Examination

After sacrifice and dissection, vital organs, including the pancreas, liver, kidneys, spleen, and heart, were carefully excised from all experimental animals and immediately fixed in 10% neutral buffered formalin for 48 h to preserve tissue architecture. The fixed samples were subsequently sent to the Department of Pathology, Doctors Lab and Hospital (Pvt.) Ltd., Kushtia, Bangladesh, for histopathology processing.

The tissues were dehydrated using graded alcohols and xylene was used for clearing, and then they were embedded in paraffin blocks. Using a rotary microtome, 5 µm thick sections were made. The sections of the pancreas were stained with a modified method of aldehyde fuchsin to study the structure of the islets and the morphology of the β-cells. Staining using H&E was performed to conduct a normal histopathological analysis of the liver, kidney, spleen, and heart specimens. Slides were stained and analyzed in a confocal microscope (model: Ti2-E; Nikon, Tokyo, Japan). Photomicrographs were drawn for comparative histological evaluation.

### 2.10. In Silico Analysis

#### 2.10.1. Selection of Bioactive Compounds

Major phytoconstituents identified through GC-MS were selected based on their relative abundance and reported pharmacological relevance to metabolic regulation. These phytochemicals’ 3D structures were retrieved from the PubChem database [29]. The ligand molecules were transformed into a pdbqt format for use in the molecular docking study after their energies had been minimized using the Open Babel tool of PyRx software (v0.8) [30,31].

#### 2.10.2. Target Protein Selection and Preparation

Target proteins associated with diabetes and thyroid regulation were selected, including thyroid hormone receptor beta (TRβ1), peroxisome proliferator-activated receptor gamma (PPARγ), and AMP-activated protein kinase (AMPK). The X-ray crystallographic structures that were recovered in the RCSB protein database had protein data bank (PDB) IDs of 1NAX, 2PRG, and 4CFF [32]. The 3D crystal structures of the proteins were assembled by removing all the heteroatoms, ligands, and water in the proteins using the BIOVIA Discovery Studio software 2021 Client (Version 21.1); then, the minimized binding energy of those receptors was saved as a PDB file to proceed with further research [33].

#### 2.10.3. Molecular Docking Analysis

The goal of molecular docking is to generate a good estimate of the structure of the ligand–receptor complex using computational methods. PyRx AutoDock Vina v1.1.2 was used to optimize and reduce the size of the proteins and ligands (compounds) before their conversion to PDBQT format [19]. The PyRx application, which also enabled the import and conversion of proteins into macromolecules, was then used to upload and convert the majority of the ligands to PDBQT format. A grid box surrounded a functional point of interest. After a PyMOL analysis yielded a complex structure, the dock file was uploaded to the BIOVIA Discovery Studio Visualizer to show protein–ligand pocket diagrams and the locations of active amino acids. The co-crystallized ligands were re-docked into the active sites of their corresponding target proteins using the same docking settings to verify the docking technique [34]. Ultimately, the BIOVIA Discovery Studio Visualizer was used to visualize the interactions in protein–ligand complexes.

### 2.11. Statistical Analysis

All experimental data were analyzed using SPSS software version 24.0 (IBM Corp., Armonk, NY, USA). Data were expressed as means ± SEMs (n = 8). Differences among multiple groups were assessed using one-way analysis of variance (ANOVA), followed by Tukey’s HSD post hoc tests. *p* < 0.05 was statistically significant, and *p* < 0.001 was highly significant.

### 2.12. Ethical Considerations

All experimental procedures involving animals were carried out in strict accordance with the institutional ethical guidelines and principles outlined in the Guide for the Care and Use of Laboratory Animals. Ethical approval for the study was obtained from the Institutional Animal Welfare and Ethical Committee of the Faculty of Biological Sciences, Islamic University, Bangladesh [Approval No.: AWECC/FOB/IU/2025 (04); Approval Date: 15 January 2025]. All efforts were made to minimize animal suffering and to reduce the number of animals used.

## 3. Results

### 3.1. Phytochemicals Identified from GC-MS Analysis of Borassus flabellifer Immature Endosperm

**Figure 1:**
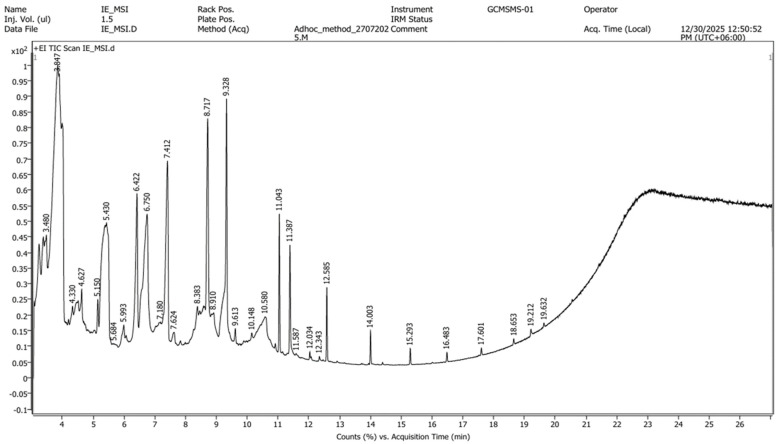
Characterization of *Borassus flabellifer* immature endosperm (BFE) using GC-MS.

The GC-MS analysis of the hydroethanolic extract of *Borassus flabellifer* immature endosperm (BFE) showed a wide variety of phytochemical components. A total of 33 unique compounds were recognized through retention time, molecular weights, and fragmentation patterns, with spectral data cross-referenced against the NIST library database for verification. The chromatographic peaks illustrate various categories of bioactive compounds, including hydrocarbons, fatty acid esters, alcohols, terpenoids, and sterols. Among the identified compounds, the most abundant compounds based on peak area (%) were D-Alanine, N-propargyloxycarbonyl-, propargyl ester (35.46%), Inositol (15.68%), Furandimethanol (7.24%), 2-Butyn-1-al diethyl acetal (7.24%), 5-Hydroxymethylfurfural (5.94%), caffeine (5.39%), 2-Pyrrolidinone, 5-(cyclohexylmethyl)- (3.37%), 2,4-Imidazolidinedione, 1-methyl- (1.63%), and 2-propylphenol (1.55%). The comprehensive GC-MS chromatogram is shown in Figure 1, and the complete compilation of identified compounds, along with their retention times, molecular weights, chemical formulas, and relative percentage peak areas (%), is outlined in Table 1. The variety and intricacy of the detected metabolites emphasize the phytochemical abundance of BFE, indicating its potential to serve as a source of pharmacologically significant natural products.

It should be noted that compound identification was made based on spectral matching with the NIST library, and this information should therefore be regarded as tentative. Confirmation of these compounds will require further characterization by complementary analytical techniques and/or authentic reference standards.

### 3.2. Modulation of Thyroid Hormone Biomarkers by Borassus flabellifer Immature Endosperm Extract

**Figure 2:**
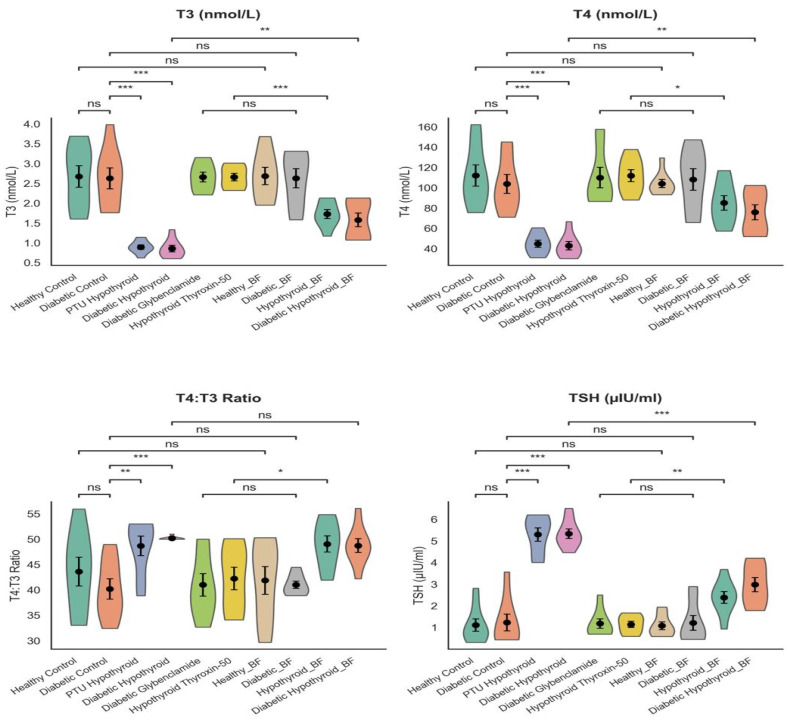
Effect of *Borassus flabellifer* endosperm (BFE) treatment on thyroid hormone biomarkers in experimental rats. Values are expressed as means ± SEMs (n = 8). Statistical differences among groups were analyzed using one-way ANOVA followed by Tukey’s HSD post hoc test. * *p* < 0.05, ** *p* < 0.001, “***” indicates highly significant, ns: non-significant.

As presented in Figure 2, it was possible to demonstrate the variation in the circulating thyroid hormones in the experimental groups. The level of triiodothyronine (T3) and thyroxine (T4) in the serum was significantly lower, and the thyroid-stimulating hormone (TSH) in the PTU and combined disease (DHC) groups rose significantly (*p* < 0.05–0.001), which is the confirmation of the successful induction of the dysfunction of the thyroid and impairment of the hypothalamic–pituitary–thyroid (HPT) axis.

Administration of *Borassus flabellifer* immature endosperm extract (BFE) resulted in a marked and dose-dependent restoration of thyroid hormone homeostasis. The levels of T3 and T4 had significantly increased in all the treated groups, and the level of TSH, which depicts a restoration of thyroid functioning, had reduced. Both the HBFE and the HTBFE groups, which were closely similar to that of the healthy control, had high levels of recovery toward control levels of the hormone. Interestingly, it should be noted that the DHBFE group also showed significant improvement, but the recovery was still slightly lower compared to the treatment of a single condition.

This can be seen to be because BFE can lead to the development of the production and the peripheral conversion of thyroid hormone and reestablish the feedback control of the HPT axis by the alteration of T3 and T4 concentrations and TSH suppression. The continuous improvements in all the treatment groups are an indicator that BFE has a probability of regulating endocrine activity in both isolated and mixed pathological conditions. The degree of normalization was comparable to that of the normal hypothyroid drug; hence, it implies that BFE can be endocrine-regulated.

Overall, Figure 2 demonstrates that BFE correlated with improved thyroid hormone profiles in this experimental model, implying a possible endocrine-modulatory activity, which should be further investigated, especially its mechanism of action.

### 3.3. Effect of BFE on Hyperglycemia

**Figure 3:**
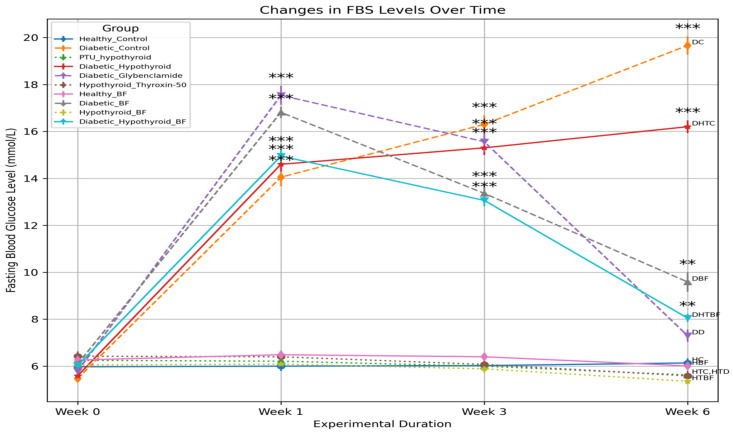
Effect of *Borassus flabellifer* endosperm (BFE) on hyperglycemia. Values are expressed as means ± SEMs (n = 8). Statistical differences among groups were analyzed using one-way ANOVA followed by Tukey’s HSD post hoc test. ** *p* < 0.001, “***” indicates highly significant.

Figure 3 reveals that fasting blood glucose (FBG) was significantly increased in the diabetic (DC) group and the combined disease (DHC) group compared with the healthy control (HC) group (*p* < 0.001), which exhibits the development of hyperglycemic conditions. The scale of increment was larger in the DHC group, which suggests worsened metabolic impairment in combined pathological conditions. The BFE treatment led to a substantial decrease in the FBG levels in all the intervention groups. The antihyperglycemic response was also strong, as the DBFE group exhibited a significant reduction in glucose levels in comparison to the diabetic control. Likewise, the DHBFE group showed tremendous improvement, but the FBG levels were slightly higher than those of the DBFE group, indicating the complexity of the integrated disease status.

Notably, the effect of BFE on glucose reduction was comparable to the effect on the recovery toward control levels in comparison with the standard antidiabetic treatment. The decrease in FBG levels was also similar in all treated groups, which suggests that glycemic regulation and metabolic control were improved after administration of BFE. Overall, Figure 3 shows that BFE could help to better control glycemia in this experimental context.

### 3.4. Restoration of Hepatic Function Biomarkers

The serum glutamic pyruvic transaminase (SGPT), serum glutamic oxaloacetic transaminase (SGOT), and alkaline phosphatase (ALP) (*p* < 0.05–0.001) levels of the diabetic control (DC) and combined disease (DHC) groups were determined and compared with those of the healthy controls among the experimental rats.

The administration of BFE was important in reducing enzyme concentrations in the livers of each of the treatment groups. The HBFE and DBFE groups were significantly normalized in terms of SGPT, SGOT, and ALP, whose levels showed no correlation with the normal values of the healthy control rats. An improvement in the HTBFE group was also recorded, and this is an indication that the changes associated with thyroidism are well covered hepatically. The activity of enzymes between the DHBFE group and the DHC group was highly dissimilar and did not return to the normal condition. This extreme and complex characteristic of dual metabolic and endocrine disorders is the only reason why such remission can be assigned. The downward arrows (↓) in the treated groups also show that the concentrations of the enzymes were lower than the concentrations of the respective disease controls; hence the hepatoprotective effect of BFE. Conversely, the upward arrows (↑) in the disease groups indicate that there exist high levels of enzyme activity that are related to hepatic injury.

In sum, the idea presented in Table 2 is evidenced by the fact that BFE can also inhibit abnormalities in hepatic enzymes and contribute to the improvement of the hepatic functioning of isolated and combined pathological processes.

### 3.5. Improvement of Renal and Cardiac Biomarkers

The effect of the *Borassus flabellifer* immature endosperm extract (BFE) on the renal function markers, creatinine, urea, and blood urea nitrogen (BUN), and cardiac biomarkers, creatine kinase (CK) and creatine kinase-MB (CK-MB), is demonstrated in Table 3.

The high changes in creatinine, urea, and BUN between the diabetic control (DC) and combined disease (DHC) groups in comparison with the healthy control is an indicator of poor renal functioning. On the same note, CK and CK-MB were also highly raised in these groups, signaling myocardial stress and potential cardiac injury. The alterations in both the renal and cardiac biomarkers were also moderate, though significant in the hypothyroid (PTU) group. BFE intervention caused a drastic reduction in all the parameters within the intervention groups. Both the HBFE and DBFE groups showed high normalization of renal biomarkers, since the scores were nearly the same as those of the healthy control, which exhibited improved renal filtration and functional recovery. The cardiac biomarkers, the concentrations of CK and CK-MB, were also reduced considerably, and that was an indicator of the reduction of myocardial trauma.

The high positive reaction of the HTBFE group was also good, and it signifies that BFE can prevent the thyroid malfunctioning of the heart and the kidneys. The biomarkers were also significantly reduced in the combined treatment group (DHBFE) compared to the DHC group, though they had not been completely normalized, presumably due to the cumulative effects of both the metabolic and endocrine abnormalities. These tendencies are also confirmed by the directional (↑ and ↓) and high values in the disease groups and the low values in the treated groups, which represent the pathological changes and the therapeutic recovery with respect to the corresponding controls. Under the conditions of this study, BFE was related to beneficial changes in renal and cardiac biomarkers, which indicated possible protective effects.

### 3.6. Amelioration of Dyslipidemia

**Figure 4:**
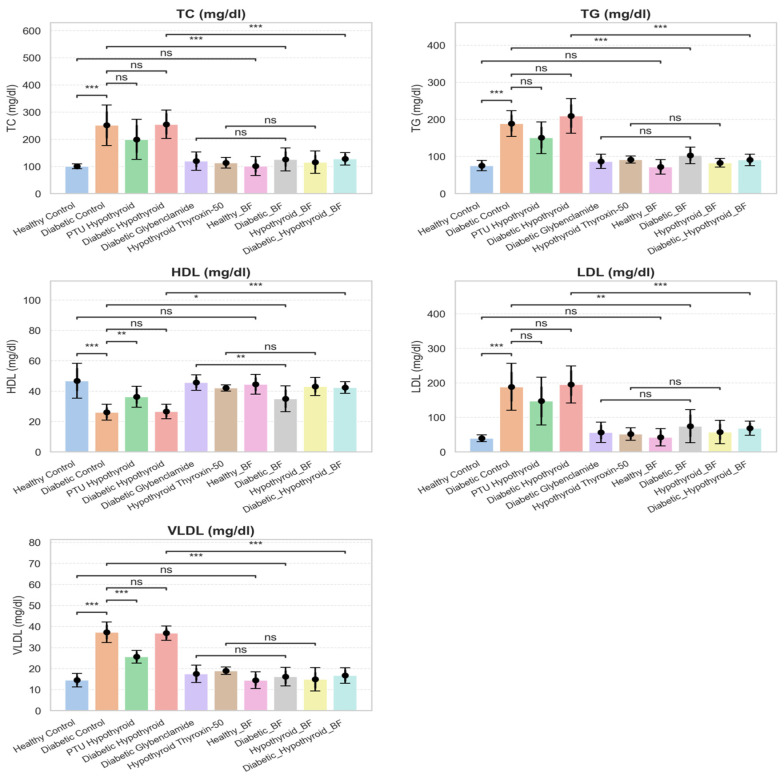
Effect of *Borassus flabellifer* endosperm (BFE) treatment on lipid profiles in experimental rats. Values are expressed as means ± SEMs (n = 8). Statistical differences among groups were analyzed using one-way ANOVA followed by Tukey’s HSD post hoc test. Treatment groups were compared with the respective control groups. * *p* < 0.05, ** *p* < 0.001, “***” indicates highly significant, ns: non-significant.

The lipid profiles of the experiment groups are shown in Figure 4. The diabetic control (DC) and combined disease (DHC) groups exhibited a highly dyslipidemic profile, in which the values changed for total cholesterol (TC) and triglycerides (TGs) and the amount of high-density lipoprotein (HDL) decreased (*p* < 0.05–0.001), which values were significant in comparison to those of the healthy control. Such changes portray the disordered lipid metabolism and the predisposition to cardiovascular complications. Moderate lipid abnormalities were also exhibited by the hypothyroid (PTU) group in accordance with the poor level of lipid clearance that was a consequence of the malfunction of the thyroid.

BFE treatment had a high impact on lipid parameters among all the intervention groups. The HBFE and DBFE groups were found to exhibit highly decreased TC, TG and LDL and increased HDL levels, with the values approximated to the normal control level. The HTBFE group, too, showed improvement, which implied that there was good modulation of thyroid-associated dyslipidemia. The mixture (DHBFE) was far more effective in causing changes in the lipid parameters, but not to normal levels, which was likely due to the multiplication of the metabolic load with the compound. However, the trend of the process of improvement in all of the treatable groups is an indicator of the lipid-regulating characteristics of BFE. The overall conclusion of Figure 4 is that the experimental model showed possible lipid-modulatory activity for BFE, as evidenced by the improvements in the lipid parameters.

### 3.7. Percentage Improvement of BFE Treatment Compared with Disease Control

Table 4 presents the extent of the biochemical parameter enhancement following the application of *Borassus flabellifer* endosperm extract (BFE) to the disease-controlling groups. The data allow for a comparative analysis of the therapeutic results of the different treatment conditions.

In terms of percentages, there were significant positive changes in all other biomarkers measured; the BFE-treated groups differed significantly, indicating that the disease controls were positive. The most significant percentage of recovery was observed in the HBFE population, in which the state of glycemic, liver (83.7), kidney (85.1), lipid (~79), and thyroid-related indicators were significantly improved (~88.4). This implies that the restorative effect of BFE is strong and applies to rather simple pathological conditions. Both the DBFE and HTBFE groups also showed a significant improvement in most of the parameters, and that shows that the metabolic abnormalities that manifest themselves with diabetes and hypothyroidism were ameliorated. Although the sizes of the changes varied for specific biomarkers, the specificity of the overall trend may indicate the constant therapeutic benefit of BFE in both single-disease models. However, the combined disease treatment (DHBFE) showed a relatively lower percentage improvement. Nevertheless, significant recovery was also seen in all the parameters, and this indicates that BFE retains biological activity even in the presence of compounded metabolic and endocrine stress. The reason why the effectiveness in this group was reduced was likely the greater severity and complexity of the dual pathological conditions.

The percentage-based analysis demonstrates the relative recovery of the physiological systems as a result of the treatment with BFE, the metabolic parameters and hepatic parameters being highly responsive to the treatment. Similar conclusions can be made in support of the widespread action of BFE and its potential concomitant modulatory impact on myriads of biological pathways. Overall, the findings in Table 4 provide the impression that BFE could result in a significant recovery of a broad spectrum of biomarkers with varying degrees of usefulness based on the complexity of the diseases, a fact that supports the possibility that BFE may influence multiple biological pathways; however, further mechanistic studies are required.

### 3.8. Therapeutic Efficacy of Borassus flabellifer Immature Endosperm Compared with Standard Drug Treatment

Table 5 contrasts the BFE and conventional drug treatment using their therapeutic efficacy as the basis. The data give the general picture of the similarity of the BFE-mediated recovery that was obtained with the assistance of the traditional pharmacological treatment.

In general, the optimal set of drugs was identified for most of the parameters and may be considered the point of reference. Interestingly, a number of the biomarkers also indicated efficacy (~95) in the HBFE group, i.e., the performance was equal to the performance of the traditional pharmacological agents. The DBFE and the HTBFE groups were also moderately highly effectual, with a robust change in the metabolic, hepatic, renal, and lipid parameters. It was not as successful as the standard drug, yet the recovery was always high, which was the measurement of the effectiveness of BFE under the diabetic and hypothyroid conditions. In the combined disease treatment group (DHBFE), it turned out to be relatively less therapeutic, and the enhancement was found to be significantly high in all the parameters measured. Such a reduced response is likely to be a result of the increased pathological burden and the metabolic and endocrine impairment. However, the fact that even in such conditions it is possible to use BFE to cause a certain amount of recovery speaks in favor of its biological activity as a multi-target intervention.

BFE does not always have the capacity to attain high pharmacotherapy levels, as disclosed in comparative analysis, though it has beneficial effects, at least in mono-disease models. This observation is especially relevant in the framework of interventions based on functional food, in which safety, accessibility, and multi-pathway modulation are benefits. In general, Table 5 shows that the biological action of BFE was confirmed in the experimental models and cannot be compared with the conventional pharmacologic treatments based on the results of the present exploratory study.

### 3.9. Percent Recovery Ranking of the General Treatment

Table 6 was prepared based on how effective each treatment was in regard to recovery of means as a percentage. This combined analysis offers a comparative profile of treatment performance in terms of the breakdown of various physiological and metabolic results into one rank structure. The reason that the standard drug group was ranked first was that it was intended as a benchmarking tool, i.e., it was expected that the levels of all the biomarkers that were studied would be constant and stable with respect to those of the other two groups (89.4). The HBFE group was found to have the highest levels among the BFE-treated groups (83.3%), which means that the therapeutic performance was better in comparatively less complicated pathological circumstances.

The DBFE and HTBFE groups were in the middle of the list (74.4 and 71.6 respectively) and were defined by the large recovery of most of the biochemical parameters. The similarity in their effects implies that BFE has a constant therapeutic effect on diabetic and hypothyroid conditions, with slight differences between the diseases. On the contrary, the DHBFE group was ranked lower (64.5%), which denoted a comparatively lower overall recovery. The second aspect that can be traced is that the integrated disease model has an augmented physiological load at which secondary failure of the metabolic and endocrine system can restrict the extent of response to treatment. The statistically significant enhancement in this group, however, proves that BFE is still functional in even more pathological cases.

The pattern of the ranking is only one of the indications that BFE can accomplish considerable recovery in numerous biological systems, but it also shows that the complexity of the illness is one of the interconnected notions, and its responsiveness to curing is another. Interestingly enough, the fact that the BFE-treated groups and especially the HBFE group were relatively high-ranking also contributes to the suggestion that the intervention can prove to be effective as a potential multi-pathway biological activity. Overall, Table 6 could be considered a concise yet comprehensive summary of the therapeutic effectiveness and justifies the potential relevance for metabolic and endocrine dysfunction of BFE and its possible application in the treatment of metabolic and endocrine disorders.

### 3.10. Inter-Organ System Metabolic Recovery Index

Table 7 displays a combination of percent metabolic recovery indices, providing a summation of the total therapeutic effect of the *Borassus flabellifer* endosperm extract (BFE) on the different organ systems—hepatic, renal, cardiac, endocrine, and metabolic parameters. It is the composite index that provides a general perspective on the treatment’s effectiveness as the aggregate of the changes that occur in the majority of the spheres of physiological functioning.

The most successful was the standard drug group in regard to the index of recovery, since it was found that it worked equally with all systems that were tested and served as a reference benchmark. The integrated recovery of the HBFE group (82.4) was best out of the BFE-treated groups, which showed a high and balanced therapeutic effect on most of the organ systems in less complicated disease conditions.

The recovery indices of both the DBFE and HTBFE groups were also significant (74.0 and 71.2, respectively), and this implies that BFE can cope with the metabolic and endocrine disturbances. The circumstance that the values of these groups are quite similar declares that BFE does not evolve in terms of its efficiency in the process of treating diabetes- and hypothyroidism-related dysfunctions. The DHBFE group, on the contrary, was relatively low in terms of recovery index (64.2%). This weakened reaction is likely to be a reflection of the augmented load of two pathological procedures, whereby there is metabolic and hormonal deregulation, which does not allow a complete manifestation of physiological restoration. It is of the utmost importance that the enhancement in the DHBFE group, however, implies that BFE could still have a certain tangible impact on the treatment of complex disease conditions.

The integrated index points out the systemic impacts of BFE, demonstrating that the impacts of this system do not pertain to a single organ or pathway but are distributed across the different interrelated biological systems. This multi-target, polypharmacological approach is consistent with the concept of multi-target, multi-spectrum recovery, which is consistent with the wide variety of phytochemical compositions observed with the assistance of GC-MS analysis. Overall, Table 7 highlights the possibility of using BFE to support the systematic metabolic repair of all body systems, the implication of which is that the modality can be employed as a multi-purpose, multi-system therapy for complex metabolic and endocrine diseases.

### 3.11. Reduction in Cardiovascular Risk Indices

Table 8 illustrates the cardiac risk indices of the experimental rats that were fed on the extract of the *Borassus flabellifer* immature endosperm (BFE). The indices measured are the atherogenic index (AI), the Castelli risk indices (CRI-I and CRI-II), the atherogenic coefficient (AC), the lipoprotein combined index (LCI), and the CK-MB/CK ratio, which provides an overall evaluation of cardiovascular risk status.

The diabetic control (DC) and combined disease (DHC) groups showed highly significant values for all cardiac risk indices as compared to the healthy control (*p* < 0.05), which means that they have a greater atherogenic potential and a higher risk of cardiovascular diseases. Such changes are indicative of lipid metabolism and cardiac stress dysregulation with respect to metabolic and endocrine disruptions. Moderate changes in various indices were also observed in the hypothyroid (PTU) group, which is in line with the inability to manage lipids and cardiovascular vulnerability.

The BFE treatment led to a considerable decrease in all the cardiac risk indices in the intervention groups. In the HBFE and DBFE groups, improvements in AI, CRI-I, CRI-II, AC, and LCI values and a decrease in the CK-MB/CK ratio reflects an improvement in lipid balance and decreased myocardial damage. These values were close to those of the healthy control, which indicated effective prevention of cardiovascular risk. The HTBFE group was also greatly enhanced, which implies that BFE decreases thyroid-associated variations in cardiovascular risk factors. All the indices were considerably lower in the combined treatment group (DHBFE) than in the DHC group, but they were not fully normalized, probably because of the compounding effects of the two pathological conditions.

The negative tendencies (↓) in the treated groups indicate the cardioprotective effect of BFE, and the presence of cardiovascular risk is proved by the high values (↑) in the disease groups. The gradual increase in the different indices is a sign that BFE can alter lipid-related and enzyme cardiac health indices. In general, Table 8 shows that BFE has a great effect in decreasing cardiovascular risk and enhancing cardiac biomarker profiles, which justifies its possible use in preventing cardiometabolic complications related to diabetes and hypothyroidism.

### 3.12. Effect of BFE on Body Weight, Food Intake, and Metabolic Index

A summary of the effects of *Borassus flabellifer* endosperm extract (BFE) on the body weight development, food intake, feed ratio (FER), and metabolic index (FBG/BW ratio) in the experimental rats is presented in Table 9.

At baseline, the initial body weights were homogeneous in all groups, and this implies that they were similar before the intervention. Nevertheless, the diabetic control (DC) and combined disease (DHC) groups showed a considerable decrease in final body weight and percentage weight gain as compared to the healthy control, although there was a comparatively higher or similar food intake. The trend is a sign of dysfunction in nutrient consumption and metabolic efficiency associated with hyperglycemia and endocrine dysfunction.

The DC and DHC groups exhibited significant reductions in the ratio of feed efficiency, which once again tended to support the hampered metabolization of dietary intake to body mass. At the same time, the metabolic index (FBG/BW ratio) in these groups was significantly higher, and this is an indication of bad glycemic control relative to body weight. These parameters significantly benefited from the administration of BFE in all the treated groups. The HBFE and DBFE groups realized a considerable increase in the end body weight and percentage weight gain as the food consumption was normalized and when the feed efficiency notably improved. The changes obtained are a sign of high intake of nutrients and metabolic recovery.

In the HTBFE group, there was also an overall improvement, which is a good indication that BFE is an effective intervention in the management of metabolic imbalances associated with thyroidism. Body weight gain, FER, and the metabolic index were also significantly better than in the DHC group in the combined treatment group (DHBFE), but full recovery toward control levels was not attained, probably because of the greater severity of combined metabolic and endocrine stress.

The fact that the FBG/BW ratio in the treated groups showed a decrease is an indicator of improved glycemic control compared to body mass, and gain (↑) in body weight gain and FER are indicators of improved metabolic efficiency.

In general, Table 9 demonstrates that BFE is an effective method for restoring body weight dynamics, enhancing the use of feed, and enhancing the percentage of metabolic stability, which can help to restore energy balance and metabolic pathology in experimental models.

### 3.13. Histopathological Evaluation of Tissue Architecture (Figure 5A–D)

**Figure 5:**
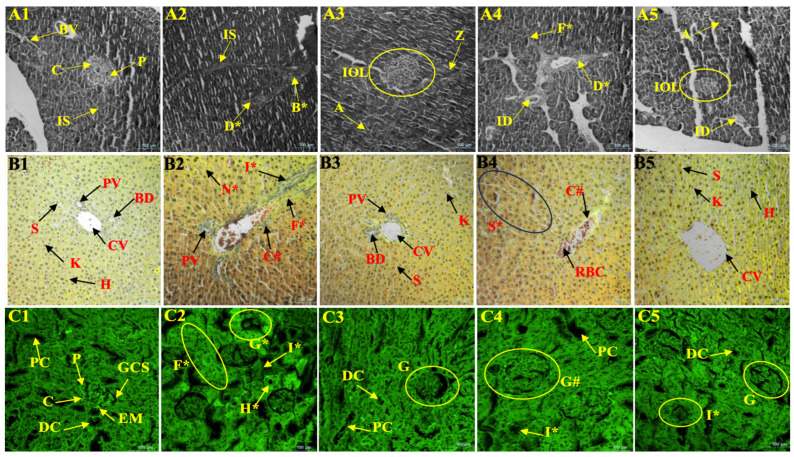

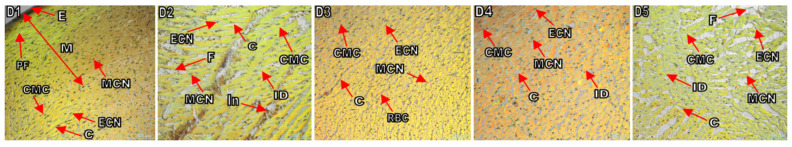
(**A**–**D**) Histopathological micrographs of pancreas, liver, kidney, and heart tissues. Representative histological sections from control and treated groups. Each row depicts stained micrographs of (**A**) pancreas, (**B**) liver, (**C**) kidney, and (**D**) heart tissues from different experimental groups and subfigures (1–5) indicates healthy control (HC), diabetic control (DC), Healthy rats treated with BFE (HBFE), Diabetic rats with glibenclamide (DD), and Diabetic rats treated with BFE (DBFE) respectively. Sections were stained and observed via confocal microscopy to illustrate changes in cellular architecture due to diabetes and treatment with (**B**) flabellifer endosperm or glibenclamide. Scale bars = 100 μm. Abbreviations: A = acini; BV = blood vessel; B* = β-cell depletion; C = capillaries; D* = ductal hyalinization; F* = fibrosis in intralobular connective tissue; PIC = pancreatic islet cell; CTC = connective tissue capsule; ID = interlobular duct; IS = interlobular septum; IOL = islet of Langerhans; Z = zymogen granules.

Figure 5 shows representative histopathological micrographs of the pancreas tissue, liver tissue, kidney tissue, and heart tissue (A–D) of the experimental groups that present the structural alterations of the disease conditions and the therapeutic effects of *Borassus flabellifer* immature endosperm extract (BFE).

Histopathological changes were assessed by a pathologist who was blind to the treatment groups and were scored independently on a semi-quantitative scale (0 to 4) based on published criteria for the assessment of tissue damage, with 0 representing normal histology and 4 severe tissue damage (Table 10).

Pancreas (Figure 5A)

The healthy control population (clear Langerhans islets and intact acinar cells) showed the presence of the normal pancreatic arch. On the other hand, islet cell degeneration, decrease in islet size, and alterations in cellular organization, which are indicators of beta-cell damage, were observed in both the diabetic control (DC) and combined disease (DHC) groups. The BFE-treated groups showed a significant restoration of pancreatic structure, the degree of restoration demonstrated by the enhancement of islet integrity and cell organization. The recovery was normal in terms of morphology in the HBFE and DBFE groups and moderate in the DHBFE group, and partial recovery occurred under the combined pathological stress.

Liver (Figure 5B)

The hepatic architecture of the liver areas in the healthy control group was normal with intact central veins, organized hepatocytes, and hepatic spaces. There were clear pathological alterations in the DC and DHC groups that included hepatocellular degeneration, sinusoidal congestion, and disorganization of cells. The BFE treatment greatly enhanced the hepatic morphology and cellular damage, and normal architecture was restored. The HBFE and DBFE groups were very much recovered, and the DHBFE group was moderately recovered.

Kidney (Figure 5C)

Kidney tissues of the healthy control group displayed the normal glomerular and tubular structures. The DC and DHC groups did not experience such changes to the interstitium, tubular necrosis, and glomerular atrophy, hence demonstrating that they had bad renal function. This resulted in a significant improvement in renal histology, as well as the restoration of the renal glomerular structure and tube integrity by the treatment with BFE. The HBFE group and the DBFE group showed the highest recovery, and the DHBFE group showed recovery that was partial but substantial.

Heart (Figure 5D)

The healthy control group’s heart tissues were characterized by normal cells and myocardial fibers. The DC and DHC groups showed ruptured myocardial fibers, cellular degeneration, and structural disorganization, which are indications of cardiac stress. The BFE-treated groups were primarily defined by an improvement in myocardial architecture and tissue alignment of fibers, and the structural injury was also recovered. The morphology of the HBFE and DBFE groups was almost normal, and the DHBFE group recovered moderately.

Overall Interpretation

Collectively, the histopathological findings confirm that the disease conditions resulted in extreme structural alterations of several organs, and BFE therapy was useful in minimizing these alterations. The result of the biochemical analysis was commensurate with the finding that the degree of recovery of tissues when using single-disease models was greater than that observed for the combined disease condition. On the whole, Figure 5 shows the morphological data evidencing that BFE has multi-organ protective properties which can be used to restore structural and functional integrity during metabolic and endocrine stress.

### 3.14. Molecular Docking Study

**Figure 6:**
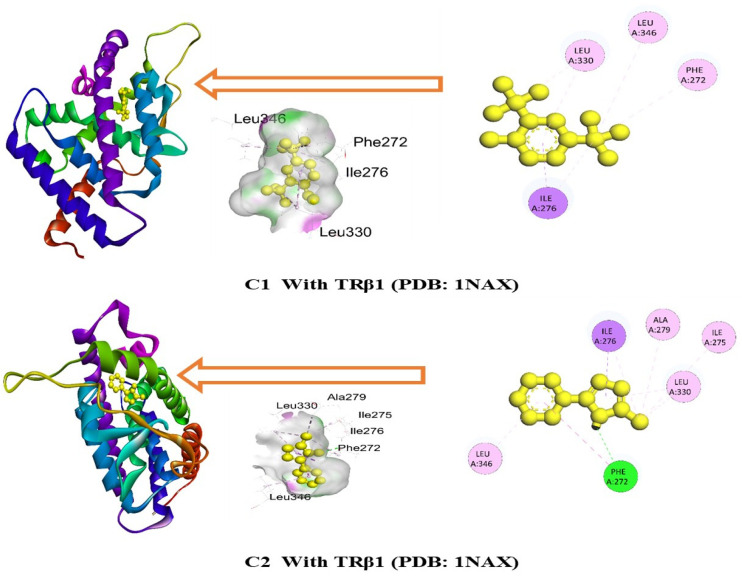
The optimum interaction between the identified ligands (C1 and C2) and the PDB: 1NAX receptors (TRβ1) within the binding pocket is depicted in 3D and 2D forms.

The blind docking study revealed that the highest binding affinity was observed for C1, with a binding affinity of −7.8 kcal/mol, followed by C2, C3, C4, and C5, which exhibited binding affinities of −7.6, −7.3, −7.1, and −7.1 kcal/mol, respectively, against the TRβ1 receptor (PDB: 1NAX) (Table 11). Figure 6 demonstrates that C1 attached to six amino acids of TRβ1 receptors (PDB: 1NAX), namely, ILE276, LEU330, ILE276, LEU346, PHE272 and LEU330, while C2 attached to nine amino acids, namely, PHE272, ILE276, PHE272, ALA279, ILE275, ILE276, LEU330, LEU330 and LEU346.

**Figure 7:**
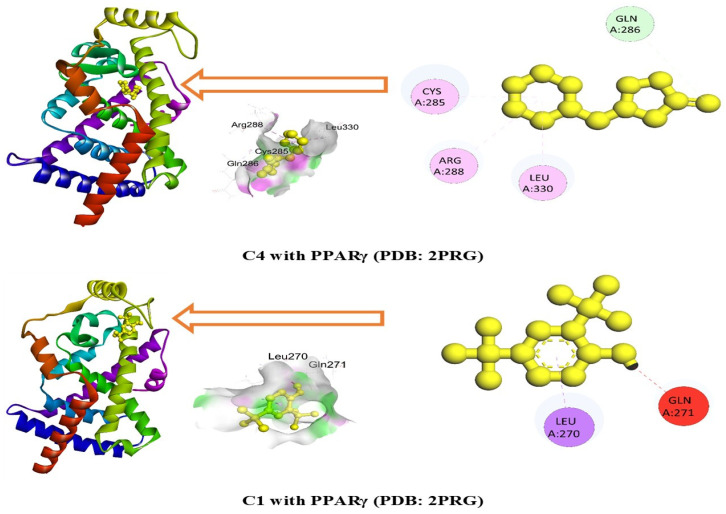
The optimum interaction between the identified ligands (C4 and C1) and the PDB: 2PRG receptors (PPARγ) within the binding pocket is depicted in 3D and 2D forms.

Table 11 and Figure 7 demonstrate that C4 exhibited notable binding affinity against the PPARγ receptor (PDB: 2PRG) through a series of bonds with the amino acids, including GLN286, CYS285, ARG288, and LEU330, with a binding score of −6.2 kcal/mol. On the other hand, C1 is bound to only one amino acid residue of the PPARγ receptor (PDB: 2PRG), namely, LEU270, and shows a binding affinity of −6.2 kcal/mol. Additionally, C22, C7, C2, and C24 showed notable binding affinities of −6.1, −6.1, −6, and −5.9 kcal/mol against the PPARγ receptor (PDB: 2PRG), respectively.

**Figure 8:**
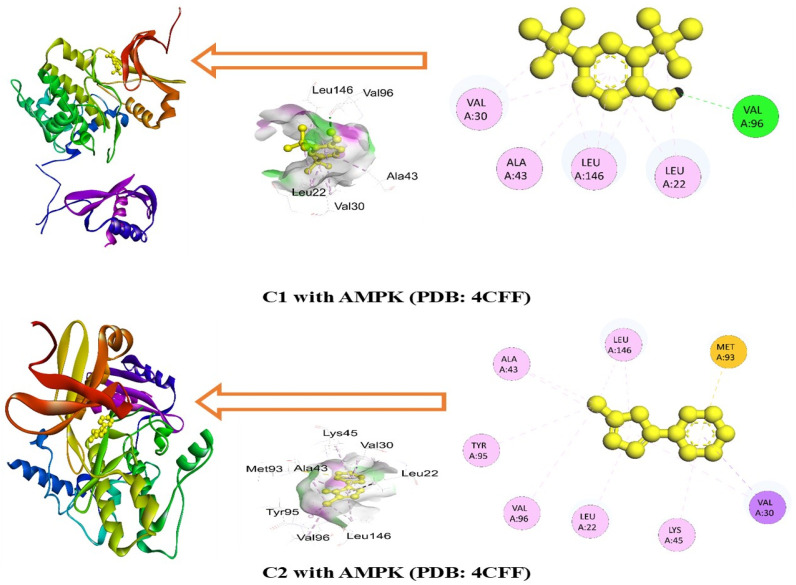
The optimum interaction between the identified ligands (C1 and C2) and the PDB: 4CFF receptors (AMPK) within the binding pocket is depicted in 3D and 2D forms.

In the case of the AMPK receptor (PDB: 4CFF), the most prominent affinity was witnessed in C1 and C2 with binding affinity of −6.7 and −6.3 kcal/mol. On the other hand, C22, C6, C4, and C16 manifested promising affinities with values of −6.2, −5.9, −5.8, and −5.8 kcal/mol, respectively. However, C1 bound to nine amino acids of the AMPK receptor, namely, VAL96, LEU22, LEU146, VAL30, LEU146, LEU22, VAL30, ALA43, and LEU146, as shown in Figure 8, while C2 bound to VAL30, ALA43, VAL96, LEU146, TYR95, LEU22, VAL30, ALA43, LEU146, LYS45, and MET93 amino acids. Amino acid residues and bond distances for the highest binding ligands, such as C1, C2, and C4, with the targeted protein are included in Table 12a–c.

## 4. Discussion

The results of the present study are to be regarded as the preliminary data of a research study that was exploratory and not designed to demonstrate a specific molecular mechanism. Concurrently evaluating the glucose, hormonal, lipid, biochemical and histopathological parameters was planned to study the coordinated effect of *Borassus flabellifer* immature endosperm extract (BFE) in interconnected physiological systems of diabetes and hypothyroidism. The present research offers much evidence that supports the idea that BFE exerts multi-target therapeutic effects on the metabolic pathology of diabetes and hypothyroidism and the blend of the two diseases. Interestingly, a combination of GC-MS phytochemical profiling, molecular docking, and in vivo data gives mechanistic explanations of the biological activity of the extract.

The previous studies on *Borassus flabellifer* were limited to its nutritional profile, antioxidant activity or specific metabolic effects [5,8]. However, this study combines an analysis of phytochemical characterization (GC-MS) with molecular docking, endocrine and metabolic biomarker assessment, cardiovascular risk analysis, and histopathological evaluation in both isolated diabetic and combined diabetic–hypothyroid disease models. To the best of our knowledge, this is the first study to explore the dual modulation of diabetes and hypothyroidism using *Borassus flabellifer* immature endosperm extract using an integrated in silico–in vivo approach.

Chromatograms and identification of compounds via GC-MS (Figure 1 and Table 1) revealed that BFE contains a chemically diverse mixture of phenolic and methylxanthines, fatty acids, heterocyclic molecules, and sugar alcohols such as inositol. This heterogeneity suggests that the biological effects that have been seen are most likely due to polypharmacological action rather than a single active constituent.

The other notable feature of the GC-MS profile is the existence of huge concentrations of phenolic derivatives such as 2, 4-di-tert-butylphenol, 4-vinylbenzene-1, 2-diol, and 4-hydroxy-2-methylacetophenone. These are familiar antioxidant and free radical scavenging agents which are significant in lowering oxidative stress, a significant cause of diabetes and thyroid dysfunction. Oxidative stress inhibits insulin signaling, kills pancreatic β-cells, and interferes with thyroid hormone synthesis [35]. The redox-modulating capacity of these phenolic compounds is, therefore, presumably related to recovery toward control levels of the biochemical parameters of the BFE-treated groups.

The identification of a compound was considered tentative, and several compounds exhibited moderate match factors when the spectral similarity matching method in the NIST library database was used, so the interpretation of the mechanism needs to be verified by advanced analytical techniques, such as LC-MS/MS or NMR.

However, the presence of 5-hydroxymethylfurfural (HMF) and furan analogues shows an extra antioxidant and cytoprotective effect. Such compounds have been reported to decrease lipid peroxidation and defend cellular macromolecules in metabolic stress states, which might be a cause of the reported improvements in hepatic and renal biomarkers.

Of special interest is the relevance of the presence of methylxanthines, caffeine, and theobromine specifically to the antihyperglycemic effect of BFE. They are claimed to increase cellular energy metabolism and insulin sensitivity (in part via activation of AMP-activated protein kinase (AMPK) and regulation of intracellular cyclic AMP signaling). This coincides with the massive decrease in fasting blood glucose and metabolic index improvement in the DBFE and DHBFE groups [36,37]. In addition, the effects of methylxanthines must be of a protective nature to pancreatic β-cells that would help in discharging insulin and glucose regulation [38]. Similarly, the improvement in pancreatic histoarchitecture is an indicator of β-cell maintenance, which may indicate that BFE can also suppress glucotoxicity-induced β-cell apoptosis. This bifurcative effect on insulin sensitivity and insulin secretion is the basis of the mechanism of glycemic improvement, which was significant, especially in the DHBFE group.

The other interesting observation after the GC-MS analysis is the presence of inositol as one of the molecules of the insulin signaling pathway. Inositol and analogs of inositol are secondary messengers of glucose intake with insulin, as well as being reported to increase insulin sensitivity and glycemic control [39]. The enhanced FBG/BW ratio and the heightened metabolic performance of the BFE-treated groups could have a plausible molecular basis due to the identification of inositol.

BFE has a lipid-modulating effect that is further supported by the identification of fatty acids and their derivatives, such as n-hexadecanoic acid, methyl stearate, and long-chain amides (Z)-13-docosenamide, among others. It was claimed that these compounds affect lipid and membrane stability and inflammatory signaling, and this could be the reason behind the great changes in lipid profile and cardiac risk indices [40]. Moreover, the peroxisome proliferator-activated receptors (PPARs), in particular, PPAR-α, are also activated by fatty acids, which enhances fatty acid oxidation and lowers the amount of triglycerides [41].

The fact that BFE treatment normalizes levels of thyroid hormones (T3, T4, and TSH) can also be credited to its phytochemical composition. Another effect has also been reported in the metabolism and activity of thyroid hormone receptors (TRα/β) and iodothyronine deiodinases (DIO1/DIO2) by heterocyclic molecules and phenolic compounds. In addition, the hepatoprotective effect of BFE may indirectly enhance the peripheral conversion of T4 to the biologically active T3, which causes the restoration of thyroid homeostasis [42,43]. The fact that the DHBFE group was capable of regaining its normal thyroid activity despite the fact that the group had a severe two-pathology disorder demonstrates a functional salvage of the hypothalamic–pituitary–thyroid (HPT) axis.

The in silico docking findings also favored this multi-target potential, as some of the identified compounds exhibited moderate binding affinities to the key proteins involved in the regulation of glucose and thyroid metabolism, including thyroid hormone receptor β (TRβ1), PPAR-γ, and AMPK. It is also worth noting that C1 and C2 were the most affine to TRβ1 with docking scores of −7.8 and −7.6 kcal/mol. Hydrogen bonding was also not the only type of binding force; hydrophobic binding forces with other residues such as Phe272, Ile276, and Leu330 were also observed, which signifies a positive binding conformation in the receptor pocket. These forces can either increase or decrease receptor stimulation or control, which increases thyroid hormone signaling. However, limited phytoconstituents showed comparatively higher predicted docking scores, so the docking should be taken as a supporting computational prediction and not as a definite proof of bioactivity, as it is likely that the observed therapeutic activity was due to the synergic effect of the multiple phytochemicals present in the sample rather than a single high-affinity ligand.

PPARγ activation and DPP4 inhibition have been observed to stimulate insulin release and incretin secretion, respectively, and to enhance insulin sensitivity and lipid metabolism [44,45]. Moreover, communication with TRβ1 suggests a potential pathway through which BFE may influence thyroid hormone signaling and metabolic rate. Such results suggest the mechanistic importance of the identified phytochemicals and support their participation in the dual regulation of metabolism.

Likewise, the contacts with PPARγ and AMPK suggest the possibility of controlling lipid metabolism and cellular energy homeostasis. These substances may be useful ligands because of the presence of hydrophobic residues and critical bonding interactions, which influence insulin sensitivity and metabolic homeostasis. This concomitant targeting of the proteins highlights a polypharmacological effect, and it is particularly advantageous in more complicated diseases with multiple dysregulated pathways. However, while several compounds showed good docking scores against some of the selected targets, the results from the computational study should be viewed as preliminary and complementary to further studies on the mechanism of action or pathway modulation and not as proof of therapeutic activity. The in vivo findings are very much supportive of these molecular insights.

Biochemical experiments in vivo were conducted as per the prediction of docking and showed that BFE reduced the concentration of fasting blood glucose to a very small extent and increased the metabolic index in the diabetic and combined disease groups. These effects may be attributed to the heightened glucose uptake and insulin sensitivity, which might be mediated by such pathways as AMPK activation and PI3K/Akt signaling, which are central to glucose homeostasis [14]. BFE exhibited an antihyperglycemic effect, which is in line with other studies that have reported the glucose-lowering effect of plant-based bioactive compounds [46].

The enhancement of the hepatic and renal biomarkers in the present study is in line with the antioxidant and anti-inflammatory properties of the identified phytochemicals combined. Chronic hyperglycemia and hypothyroidism, which both contribute to tissue damage, are associated with activation of reactive oxygen species (ROS) and inhibition of NF-κB-mediated inflammatory signaling [16]. BFE will tend to maintain hepatocyte integrity by inhibiting oxidative and inflammatory stress that ultimately results in lipid metabolism and conversion of thyroid hormones. The interaction demonstrates the critical role of the liver as a metabolic center between the endocrine system and glycemic control. Similarly, the partial restoration of renal biomarkers (creatinine, urea, and BUN) is a sign that glomerular and tubular damage is protected. Renal oxidative stress and microvascular dysfunction are proven to be induced by chronic hyperglycemia and hypothyroidism. These enhancements show that BFE has a potential impact of increasing the antioxidant capabilities of the kidneys and the activity of microcirculation and, therefore, maintenance of the filtration capacity and restoration of histological structure.

Among the most important characteristics of this work, the correction of dyslipidemia and the reduction in the cardiac risk indices are to be mentioned. The radical decrease in TC, TGs, AI, CRI, AC, and LCI suggests that BFE changes the lipid metabolism at different levels. PPAR-α stimulation resulting in the burning of fatty acids and a decrease in triglyceride levels [47] is one of the most probable pathways. Furthermore, a better thyroid hormone condition increases LDL receptor expression and cholesterol ejection, which also improves lipid levels toward control levels. Myocardial injury is attenuated by a decrease in CK and CK-MB levels and could be caused by a decrease in lipid peroxidation and stabilization of cardiomyocyte membranes.

In this study, biochemical and metabolic parameter recovery rates were also compared with respect to conventional pharmacological therapy and BFE. Table 5 and Table 6 were constructed to allow for a general overall evaluation of the overall treatment-mediated recovery patterns and to see how much the improvement in the treatment-mediated recovery patterns was similar to that obtained by standard drug therapy. The main reference standard was glibenclamide, since most of the evaluated parameters were related to diabetic and/or cardiometabolic dysfunction. L-thyroxine is used in the treatment of hypothyroidism, but it is used, primarily, as a hormone replacement therapy and not for general modulation of metabolism. Therefore, one cannot make unequivocal comparisons of the effectiveness on all measures. This is a limitation of the present comparative analysis and should be taken into consideration when interpreting the results.

The histopathological micrographs and blinded semi-quantitative histopathological scoring approach also provide biochemical evidence as they show pancreatic, hepatic, renal, and cardiac tissue structural recovery (Table 10, Figure 5). It is implied that pancreatic islet preservation prevents oxidative and inflammatory damage, which is probably due to the phenolic antioxidants and metabolic support by methylxanthine. Likewise, better hepatic and renal morphology indicates less cellular degeneration and a better repair process.

The recovery of body weight gain and feed efficiency ratio (FER) also helps in the restoration of metabolic homeostasis. The inability to transform energy in the mitochondria and a high rate of catabolism could be considered the manifestation of the low FER in the disease conditions without any treatment. BFE greatly increased FER metabolism, and one possible explanation is that there was an increased rate of oxidative phosphorylation and ATP production in the mitochondria, which would have occurred due to the action of AMPK activation and inhibition of oxidative stress. This is in line with the new evidence that phytochemicals have the potential to enhance mitochondrial biogenesis and metabolic plasticity, which restores energy balance [48].

It needs to be stated that BFE demonstrated an important therapeutic effect on all the treated groups, although the level of recovery was different depending on the severity of the disease. The comparative decrease in the response of the combined disease (DHBFE) group may be due to the cumulative effects of the metabolic and endocrine malfunction. However, the observed improvements across multiple biochemical and histopathological parameters suggest that BFE may influence several biological pathways simultaneously. Nevertheless, the underlying mechanisms remain to be elucidated and require further investigation.

The antihyperglycemic and endocrine-modulatory activities observed in the present study are comparable to those observed in experimental models of plant-based nutraceuticals like *Moringa oleifera*, *Trigonella foenum-graecum*, *Gymnema sylvestre* and *Withania somnifera*, which are reported to exert similar effects on glucose metabolism, lipid homeostasis, and thyroid function [49,50,51,52]. The multi-system regulation effects of BFE, including glycemic control, lipid modulation, protection of the liver and restoration of thyroid hormone balance, were comparable to those of botanical interventions.

In the present study, the metabolic recovery of hepatic, renal, endocrine and cardiovascular biomarkers was found to be greater with immature endosperm than in the previous studies carried out with the fruit pulp extracts of *Borassus flabellifer* and the sap extracts of fruit and leaves of *B. rigidiusculus*. This can be explained by the various phytochemicals detected by GC-MS, such as inositol derivatives, phenols, methylxanthines and fatty acid esters.

In general, the analysis of the GC-MS data and in vivo data synthesis confirms the idea of a mechanism-based model, according to which BFE is able to develop its effect by polypharmacological interaction and by the concurrent expression of numerous molecular processes. This multi-layered mechanism demonstrates that the application of phytochemical-enriched extracts could be useful in the management of complex metabolic diseases, in which single-target treatment may be ineffective [53].

The present study provides preliminary evidence that BFE is associated with improvements in thyroid hormone profiles, glycemic control, lipid parameters, and selected markers of organ function in experimental models. Given the exploratory nature of the study, these findings should be considered hypothesis-generating and warrant confirmation through larger and more focused mechanistic investigations. The results of this exploratory study should be understood in the light of the study design, which includes several disease models and outcome measures. Sample sizes are comparable to those used in similar studies in preclinical research, although the statistical power of some comparisons of subgroups might be limited. So, observed effects need to be viewed as hypothesis-generating and should be followed up in larger, more specific studies.

Wider phytochemical characterization and systematic validation of molecular mechanisms is recommended for future research to pinpoint the bioactive compounds behind the effects observed and elucidate the molecular mechanisms responsible for them. These activities will improve the scientific rigor, translational relevance and therapeutic insights of BFE.

Overall, the biochemical, histopathological and computational results suggest that BFE has biological potential in experimental diabetes and hypothyroidism. Further mechanistic studies and well-powered confirmatory studies are required to draw inferences about efficacy or clinical application.

## 5. Conclusions

The present study demonstrates that the *Borassus flabellifer* immature endosperm extract (BFE) possesses multifunctional bioactivity for treating the metabolic disorder associated with diabetes and hypothyroidism. The GC-MS profiling has shown a variable combination of bioactive substances, such as phenolics, methylxanthines, fatty acids, and inositol, which sustain the biological activities. The results of molecular docking confirm the diversity of phytochemicals (according to GC-MS) with strong and stable interactions of major compounds with TRβ1, PPARγ, and AMPK, which are the primary endocrine and metabolic regulators. These molecular interactions are manifested in the in vivo results, where BFE was found to be useful in enhancements of glycemic control, thyroid homeostasis, lipid panels, and hepato-renal performance, as well as the improvement of cardiac risk factors and recovery of tissue structure. The similarity in the biochemical and histological effects indicates that it has a multi-target mode of action, which is most likely based on antioxidant actions and modulation of metabolic and endocrine processes. Even though the therapeutic response was milder in the combined disease conditions, BFE still showed a significant efficacy, which shows that it has potential for application in complex metabolic disorders. In general, the present results provide preliminary indications that BFE can have a beneficial effect on several biochemical markers, as well as histopathological and computational markers, associated with metabolic and endocrine dysfunction, and warrant further investigation in larger and more targeted studies focusing on the isolation and mechanistic validation of bioactive compounds and their clinical appraisal to guarantee increased use of bioactive compounds.

## Figures and Tables

**Table 1:** Phytochemicals in the hydroethanolic extract of *Borassus flabellifer* immature endosperm (BFE).

SL No	Component RT	Compound Name	Match Factor	Fragment Ions *m*/*z*	Formula	Molecular Weight	% Peak Area
C1	3.271	2,4-Imidazolidinedione, 1-methyl-	71.53	42.0, 43.0, 58.0, 100.0, 114.0	C_4_H_6_N_2_O_2_	114.043	1.63%
C2	3.480	D-Alanine, N-propargyloxycarbonyl-, propargyl ester	71.98	43.0, 55.0, 66.0, 83.0, 126.0	C_10_H_11_NO_4_	209.069	35.46%
C3	3.847	2-(Diethylamino)acetonitrile	74.69	41.2, 42.2, 69.1, 70.1, 97.1	C_6_H_12_N_2_	112.1	0.37%
C4	4.330	2-Pyrrolidinone, 5-(cyclohexylmethyl)-	74.28	55.1, 56.1, 83.1, 84.1, 95.1	C_11_H_19_NO	181.147	3.37%
C5	4.510	Furandimethanol	71.44	69.0, 84.0, 97.0, 109.0, 128.0	C_6_H_8_O_3_	128.047	7.24%
C6	4.511	2-Butyn-1-al diethyl acetal	72.58	65.1, 69.1, 80.1, 83.1, 97.1	C_8_H_14_O_2_	142.099	7.24%
C7	4.627	5-Hydroxymethylfurfural	80.43	41.0, 69.0, 97.0, 125.0, 126.0	C_6_H_6_O_3_	126.032	5.94%
C8	4.715	Ketone, methyl 2-methyl-1,3-oxothiolan-2-yl	72.86	43.0, 61.0, 72.0, 85.0, 103.0	C_6_H_10_O_2_S	146.04	0.65%
C9	4.739	2-Acetyl-2-methyltetrahydrofuran	80.85	43.2, 44.2, 57.1, 85.1, 87.1	C_7_H_12_O_2_	128.084	0.60%
C10	5.430	4-Hydroxy-2-methylacetophenone	80.94	77.0, 99.0, 107.0, 135.0, 150.0	C_9_H_10_O_2_	150.068	0.16%
C11	6.064	2-propylphenol	80.95	77.0, 78.0, 107.0, 108.0, 136.0	C_9_H_12_O	136.089	1.55%
C12	6.422	1-(4-methoxyphenyl)-2-Propanone	72.03	78.0, 121.0, 122.0, 128.0, 164.0	C_10_H_12_O_2_	164.084	0.78%
C13	6.750	4-Vinylbenzene-1,2-diol	73.11	89.0, 90.1, 107.1, 110.1, 136.1	C_8_H_8_O_2_	136.052	0.61%
C14	7.412	N-[2-(1H-Indol-3-yl)ethyl]hexadecanamide	78.29	95.0, 130.0, 143.0, 144.0, 151.0	C_26_H_42_N_2_O	398.33	0.83%
C15	7.624	2,4-Di-tert-butylphenol	86.03	73.0, 91.0, 191.0, 192.0, 206.0	C_14_H_22_O	206.167	1.15%
C16	8.383	1-(4-Methoxyphenyl)propane-1,2-diol	84.44	77.0, 94.0, 109.0, 121.0, 137.0	C_10_H_14_O_3_	182.094	0.95%
C17	8.422	3-Pentanone	71.80	56.1, 57.1, 72.1, 86.1, 142.1	C_5_H_10_O	86.073	0.23%
C18	8.582	3-Amino-s-triazole	71.39	43.2, 56.1, 57.1, 72.1, 84.1	C_2_H_4_N_4_	84.044	1.01%
C19	8.717	Carbamic acid, methylphenyl-, ethyl ester	80.07	79.0, 106.0, 120.0, 134.0, 179.0	C_10_H_13_NO_2_	179.095	7.03%
C20	8.910	Benzeneacetic acid, 4-hydroxy-, methyl ester	76.43	74.0, 77.0, 102.0, 107.0, 166.0	C_9_H_10_O_3_	166.063	0.36%
C21	9.072	Pyrrole, 2-methyl-5-phenyl-	77.51	115.0, 128.0, 129.0, 156.0, 157.0	C_11_H_11_N	157.089	0.05%
C22	9.328	Propanoic acid, anhydride	84.37	57.1, 58.1, 125.1, 128.1, 146.1	C_6_H_10_O_3_	130.063	0.58%
C23	9.424	Benzenesulfonamide, 4-methyl-	77.73	65.0, 91.0, 107.0, 108.0, 171.0	C_7_H_9_NO_2_S	171.035	0.21%
C24	9.588	2,5-Dihydroxy-4-methoxyacetophenone	80.14	164.1, 167.1, 168.1, 182.1, 183.1	C_9_H_10_O_4_	182.058	0.08%
C25	9.613	Decyl acrylate	82.71	55.1, 69.1, 73.1, 83.1, 97.1	C_13_H_24_O_2_	212.178	0.22%
C26	10.580	Inositol	77.20	60.1, 71.1, 73.1, 74.1, 102.1	C_6_H_12_O_6_	180.063	15.68%
C27	10.743	Hexane, 3,3-dimethyl-	81.61	43.2, 57.1, 70.1, 71.1, 85.1	C_8_H_18_	114.141	0.04%
C28	11.387	Caffeine	95.46	55.1, 67.1, 109.1, 193.1, 194.1	C_8_H_10_N_4_O_2_	194.08	5.39%
C29	11.587	Theobromine	80.02	67.0, 82.0, 109.0, 179.0, 180.0	C_7_H_8_N_4_O_2_	180.065	0.17%
C30	12.033	Pentadecanoic acid, 14-methyl-, methyl ester	89.93	43.0, 74.0, 75.0, 87.0, 143.0	C_17_H_34_O_2_	270.256	0.19%
C31	12.585	n-Hexadecanoic acid	80.68	69.1, 73.1, 83.1, 87.1, 129.1	C_16_H_32_O_2_	256.24	0.09%
C32	13.939	Methyl stearate	73.76	43.0, 55.0, 74.0, 75.0, 87.0	C_19_H_38_O_2_	298.287	0.04%
C33	19.212	13-Docosenamide, (Z)-	73.76	67.1, 69.1, 72.1, 81.1, 83.1	C_22_H_43_NO	337.334	0.09%

**Table 2:** Effect of *Borassus flabellifer* endosperm (BFE) treatment on liver function biomarkers in experimental rats.

Group	SGPT (U/L)	SGOT (U/L)	ALP (U/L)
HC	83.36 ± 7.01 ^e^	106.78 ± 5.86 ^e^	136.96 ± 19.61 ^c^
DC	251.86 ± 23.56 ^a^ ↑**	229.30 ± 22.01 ^b^ ↑**	193.38 ± 20.64 ^a^ ↑*
PTU	120.43 ± 10.28 ^c^ ↑*	135.58 ± 11.54 ^d^ ↑*	99.93 ± 3.21 ^d^ ↓
DHC	156.63 ± 11.27 ^b^ ↑**	313.79 ± 12.11 ^a^ ↑**	159.32 ± 8.50 ^b^ ↑
DD	90.98 ± 10.19 ^e^ ns	119.23 ± 11.23 ^e^ ns	103.85 ± 11.25 ^d^ ns
HTD	103.96 ± 14.07 ^d^ ns	134.44 ± 9.20 ^d^ ↑	85.49 ± 4.39 ^d^ ↓
HBFE	91.91 ± 5.73 ^e^ ns	112.78 ± 5.76 ^e^ ns	86.12 ± 10.49 ^d^ ↓
DBFE	101.33 ± 11.27 ^d^ ↑	133.53 ± 10.26 ^d^ ↑	95.72 ± 1.95 ^d^ ns
HTBFE	105.88 ± 11.52 ^d^ ↑	132.68 ± 10.59 ^d^ ↑	85.02 ± 12.38 ^d^ ↓
DHBFE	105.95 ± 12.63 ^d^ ↑	157.43 ± 12.22 ^c^ ↑*	116.54 ± 6.48 ^c^ ns

Values are expressed as means ± SEMs (n = 8). Statistical differences among groups were analyzed using one-way ANOVA followed by Tukey’s HSD post hoc test. ↑ increase; ↓ decrease compared with the respective control groups. * *p* < 0.05, ** *p* < 0.001, ns: non-significant. Different superscript letters (a–e) within a column indicate statistically significant differences among groups according to Tukey’s HSD post hoc test (*p* < 0.05). Means sharing the same letter are not significantly different.

**Table 3:** Effect of *Borassus flabellifer* endosperm (BFE) treatment on renal function and cardiac biomarkers in experimental rats.

Group	Creatinine (mg/dL)	Urea (mg/dL)	BUN (mg/dL)	CK (U/L)	CK-MB (U/L)
HC	0.57 ± 0.04 ^e^	31.68 ± 2.19 ^e^	14.80 ± 1.02 ^e^	371.85 ± 28.52 ^e^	62.41 ± 4.68 ^e^
DC	1.27 ± 0.09 ^a^ ↑**	67.84 ± 4.62 ^a^ ↑**	31.69 ± 2.16 ^a^ ↑**	682.91 ± 48.13 ^a^ ↑**	151.73 ± 11.22 ^a^ ↑**
PTU	0.75 ± 0.05 ^c^ ↑*	41.93 ± 3.11 ^c^ ↑*	19.56 ± 1.45 ^c^ ↑*	455.77 ± 32.64 ^c^ ↑*	83.54 ± 6.08 ^c^ ↑*
DHC	1.09 ± 0.06 ^b^ ↑**	56.81 ± 3.42 ^b^ ↑**	26.43 ± 1.59 ^b^ ↑**	590.48 ± 41.77 ^b^ ↑**	131.62 ± 9.54 ^b^ ↑**
DD	0.66 ± 0.05 ^d^ ns	35.98 ± 2.41 ^d^ ns	16.80 ± 1.12 ^d^ ns	398.26 ± 29.33 ^d^ ns	71.89 ± 5.02 ^d^ ns
HTD	0.70 ± 0.04 ^d^ ns	39.23 ± 2.63 ^c^ ↑	18.32 ± 1.23 ^c^ ↑	423.62 ± 31.18 ^c^ ↑	77.55 ± 5.32 ^c^ ↑
HBFE	0.64 ± 0.03 ^d^ ns	34.81 ± 2.07 ^d^ ns	16.25 ± 0.97 ^d^ ns	389.88 ± 28.06 ^d^ ns	69.21 ± 4.79 ^d^ ns
DBFE	0.69 ± 0.05 ^d^ ns	38.54 ± 2.58 ^c^ ↑	17.98 ± 1.20 ^c^ ns	417.72 ± 30.17 ^c^ ↑	74.73 ± 5.14 ^c^ ↑
HTBFE	0.72 ± 0.05 ^c^ ↑	40.22 ± 2.64 ^c^ ↑*	18.76 ± 1.24 ^c^ ↑	426.48 ± 30.81 ^c^ ↑	75.11 ± 5.28 ^c^ ↑
DHBFE	0.79 ± 0.06 ^c^ ↑*	44.08 ± 2.98 ^c^ ↑*	20.58 ± 1.39 ^c^ ↑*	448.57 ± 32.45 ^c^ ↑*	83.36 ± 5.84 ^c^ ↑*

Values are expressed as means ± SEMs (n = 8). Statistical differences among groups were analyzed using one-way ANOVA followed by Tukey’s HSD post hoc test. ↑ increase. * *p* < 0.05, ** *p* < 0.001, ns: non-significant. Different superscript letters (a–e) within a column indicate statistically significant differences among groups according to Tukey’s HSD post hoc test (*p* < 0.05). Means sharing the same letter are not significantly different.

**Table 4:** Percentage improvement of *Borassus flabellifer* endosperm (BFE) treatment compared with disease control.

Parameter	DC	HBFE	DBFE	HTBFE	DHBFE
SGPT	251.86	91.91 (83.7%)	101.33 (78.2%)	105.88 (75.3%)	105.95 (75.3%)
SGOT	229.30	112.78 (78.6%)	133.53 (66.5%)	132.68 (67.0%)	157.43 (52.6%)
Creatinine	1.27	0.64 (85.1%)	0.69 (80.1%)	0.72 (77.1%)	0.79 (71.2%)
Total Cholesterol	176.71	96.54 (78.8%)	104.74 (70.3%)	107.14 (67.7%)	111.69 (62.9%)
Triglyceride	164.52	84.27 (79.2%)	91.55 (71.8%)	93.37 (69.8%)	98.43 (64.3%)
TSH	3.84	1.72 (88.4%)	2.06 (74.7%)	2.14 (70.4%)	2.33 (60.5%)

Values in parentheses represent percentage recovery toward the normal control.

**Table 5:** Therapeutic efficacy of *Borassus flabellifer* immature endosperm (BFE) compared with standard drug treatment.

Parameter	Drug (DD)	HBFE	DBFE	HTBFE	DHBFE
SGPT	90.98	96.8%	92.5%	90.6%	90.5%
SGOT	119.23	95.2%	83.4%	84.0%	70.3%
Creatinine	0.66	97.0%	92.8%	89.4%	83.2%
TC	94.61	97.5%	90.4%	88.3%	83.5%
TGs	82.11	96.8%	90.1%	88.5%	82.7%
TSH	1.87	96.5%	84.3%	81.7%	72.9%

Values are expressed as percentage recovery relative to the disease control group. Percentage improvement was calculated relative to healthy control normalization, whereas therapeutic efficacy was calculated relative to the standard drug response.

**Table 6:** Overall therapeutic ranking of treatments based on the mean percentage recovery of biochemical parameters.

Treatment Group	Liver Marker Recovery (%)	Renal Marker Recovery (%)	Lipid Profile Recovery (%)	Thyroid Profile Recovery (%)	Overall Recovery Index (%)	Rank
HBFE	81.1	85.1	78.5	88.4	83.3	1
DBFE	72.8	80.1	70.1	74.7	74.4	2
HTBFE	71.0	77.1	67.9	70.4	71.6	3
DHBFE	63.9	71.2	62.3	60.5	64.5	4
Drug (Glibenclamide)	88.6	90.3	86.7	92.1	89.4	Reference

**Table 7:** Integrated metabolic recovery index (%) across organ systems.

Treatment	Liver	Kidney	Heart	Lipid	Thyroid	Composite Recovery (%)
HBFE	81	85	79	79	88	82.4
DBFE	73	80	72	70	75	74.0
HTBFE	71	77	70	68	70	71.2
DHBFE	64	71	63	62	61	64.2
Standard Drug (Glibenclamide)	89	90	87	87	92	89.0

The metabolic recovery index represents the mean percentage improvement in biochemical parameters relative to the disease control group. Organ system scores were calculated by averaging the recovery percentages of biomarkers within each physiological category.

**Table 8:** Cardiac risk indices in experimental rats treated with *Borassus flabellifer* endosperm (500 mg/kg, 6 weeks, n = 8).

Group	Atherogenic Index (AI) (log 10 TG/HDL)	Castelli’s Risk Index I (CRI-I) (TC/HDL)	Castelli’s Risk Index II (CRI-II) (LDL/HDL)	Atherogenic Coefficient (AC) ((TC − HDL)/HDL)	Lipoprotein Combined Index (LCI) ((TC × TG × LDL)/HDL)	CK-MB/CK Ratio
HC	0.18 ± 0.02 ^a^	2.10 ± 0.15 ^a^	0.90 ± 0.11 ^a^	1.10 ± 0.15 ^a^	1.25 ± 0.21 ^a^	0.18 ± 0.02 ^a^
DC	0.62 ± 0.05 ^c^↑	6.85 ± 1.01 ^c^↑	7.35 ± 0.91 ^c^↑	5.85 ± 1.01 ^c^↑	8.92 ± 1.34 ^c^↑	0.42 ± 0.04 ^c^↑
PTU	0.51 ± 0.04 ^b^↑	5.12 ± 1.04 ^b^↑	4.39 ± 0.92 ^b^↑	4.12 ± 1.04 ^b^↑	6.54 ± 1.21 ^b^↑	0.36 ± 0.03 ^b^↑
DHC	0.68 ± 0.06 ^d^↑	7.12 ± 1.01 ^d^↑	7.58 ± 0.92 ^d^↑	6.12 ± 1.01 ^d^↑	9.48 ± 1.41 ^d^↑	0.48 ± 0.05 ^d^↑
DD	0.22 ± 0.03 ^ab^↓	2.45 ± 0.25 ^ab^↓	1.24 ± 0.22 ^ab^↓	1.45 ± 0.25 ^ab^↓	1.82 ± 0.32 ^ab^↓	0.22 ± 0.03 ^ab^↓
HTD	0.20 ± 0.02 ^ab^↓	2.36 ± 0.17 ^ab^↓	1.22 ± 0.15 ^ab^↓	1.36 ± 0.17 ^ab^↓	1.75 ± 0.28 ^ab^↓	0.21 ± 0.02 ^ab^↓
HBFE	0.17 ± 0.02 ^a^	2.08 ± 0.17 ^a^	0.91 ± 0.15 ^a^	1.08 ± 0.17 ^a^	1.20 ± 0.19 ^a^	0.17 ± 0.02 ^a^
DBFE	0.31 ± 0.04 ^b^↓	2.78 ± 0.74 ^b^↓	2.48 ± 0.71 ^b^↓	1.78 ± 0.74 ^b^↓	3.25 ± 0.88 ^b^↓	0.26 ± 0.03 ^b^↓
HTBFE	0.24 ± 0.03 ^b^↓	2.52 ± 0.21 ^b^↓	1.28 ± 0.21 ^b^↓	1.52 ± 0.21 ^b^↓	2.10 ± 0.35 ^b^↓	0.23 ± 0.02 ^b^↓
DHBFE	0.26 ± 0.03 ^ab^↓↓	2.31 ± 0.16 ^ab^↓	1.62 ± 0.15 ^ab^↓	1.31 ± 0.16 ^ab^↓	2.05 ± 0.41 ^ab^↓↓	0.24 ± 0.03 ^ab^↓

Values are expressed as means ± SEMs (n = 8). Statistical differences among groups were analyzed using one-way ANOVA followed by Tukey’s HSD post hoc test. Different superscript letters in a column indicate significant differences (*p* < 0.05). ↑ increase; ↓ decrease compared with the respective control group.

**Table 9:** Effect of *Borassus flabellifer* endosperm on body weight, food intake, and metabolic index in experimental rats.

Group	Initial BW (g)	Final BW (g)	ΔBW (%)	Food Intake (g/d)	Total Food Intake (g)	Feed Efficiency Ratio	FBG/BW Ratio
HC	181.00 ± 1.82 ^a^	230.63 ± 2.64 ^a^	27.42 ± 1.21 ^a^	18.12 ± 0.31 ^a^	761.25 ± 12.47 ^a^	0.065 ± 0.003 ^a^	0.0268 ± 0.0016 ^a^
DC	181.13 ± 1.66 ^a^	190.25 ± 3.12 ^c^↓	5.04 ± 0.92 ^c^↓	22.31 ± 0.52 ^c^↑	937.12 ± 18.21 ^c^↑	0.010 ± 0.002 ^c^↓	0.0457 ± 0.0074 ^c^↑
PTU	183.81 ± 1.74 ^a^	197.88 ± 2.95 ^b^↓	7.63 ± 0.85 ^b^↓	20.44 ± 0.41 ^b^↑	858.50 ± 16.02 ^b^↑	0.016 ± 0.002 ^b^↓	0.0362 ± 0.0006 ^b^↑
DHC	183.25 ± 1.69 ^a^	187.75 ± 3.48 ^d^↓↓	2.46 ± 0.78 ^d^↓↓	23.05 ± 0.57 ^d^↑	968.10 ± 19.34 ^d^↑	0.005 ± 0.001 ^d^↓↓	0.0498 ± 0.0035 ^d^↑
DD	181.63 ± 1.71 ^a^	222.38 ± 2.88 ^ab^↑	22.43 ± 1.02 ^ab^↑	19.21 ± 0.38 ^ab^	806.82 ± 14.95 ^ab^	0.051 ± 0.003 ^ab^↑	0.0294 ± 0.0017 ^ab^↓
HTD	184.14 ± 1.68 ^a^	220.13 ± 2.76 ^ab^↑	19.53 ± 0.97 ^ab^↑	19.02 ± 0.35 ^ab^	798.90 ± 13.88 ^ab^	0.048 ± 0.002 ^ab^↑	0.0286 ± 0.0010 ^ab^↓
HBFE	181.44 ± 1.73 ^a^	229.75 ± 2.61 ^a^	26.63 ± 1.15 ^a^	18.05 ± 0.29 ^a^	758.25 ± 12.02 ^a^	0.064 ± 0.003 ^a^	0.0271 ± 0.0009 ^a^
DBFE	182.75 ± 1.69 ^a^	214.38 ± 2.94 ^b^↑	17.30 ± 0.98 ^b^↑	20.32 ± 0.43 ^b^	853.44 ± 15.73 ^b^	0.037 ± 0.002 ^b^↑	0.0316 ± 0.0017 ^b^↓
HTBFE	183.63 ± 1.72 ^a^	217.00 ± 2.85 ^b^↑	18.18 ± 1.01 ^b^↑	19.78 ± 0.39 ^b^	830.76 ± 14.84 ^b^	0.041 ± 0.002 ^b^↑	0.0302 ± 0.0018 ^b^↓
DHBFE	183.00 ± 1.70 ^a^	210.88 ± 3.05 ^b^↑	15.23 ± 1.04 ^b^↑	20.85 ± 0.45 ^b^	875.70 ± 16.92 ^b^	0.032 ± 0.002 ^b^↑	0.0328 ± 0.0013 ^b^↓

Values are expressed as means ± SEMs (n = 8). Statistical differences among groups were analyzed using one-way ANOVA followed by Tukey’s HSD post hoc test. Different superscript letters in a column indicate significant differences (*p* < 0.05). ↑ increase; ↓ decrease compared with the respective control groups.

**Table 10:** Semi-quantitative histopathological scoring analysis of pancreatic, hepatic, renal, and cardiac tissues in experimental rats.

Groups	Pancreas Score	Liver Score	Kidney Score	Heart Score
HC	0.25 ± 0.12 ^a^	0.30 ± 0.10 ^a^	0.20 ± 0.08 ^a^	0.22 ± 0.09 ^a^
HTC	1.35 ± 0.18 ^b^	1.62 ± 0.21 ^b^	1.48 ± 0.17 ^b^	1.40 ± 0.15 ^b^
DC	3.65 ± 0.24 ^d^	3.42 ± 0.22 ^d^	3.38 ± 0.20 ^d^	3.25 ± 0.18 ^d^
DHC	3.88 ± 0.20 ^e^	3.76 ± 0.18 ^e^	3.71 ± 0.21 ^e^	3.60 ± 0.19 ^e^
HBFE	0.42 ± 0.11 ^a^	0.48 ± 0.12 ^a^	0.45 ± 0.10 ^a^	0.40 ± 0.11 ^a^
HTBFE	1.05 ± 0.16 ^bc^	1.18 ± 0.17 ^bc^	1.10 ± 0.15 ^bc^	1.08 ± 0.13 ^bc^
HTD	0.82 ± 0.14 ^b^	0.95 ± 0.13 ^b^	0.90 ± 0.12 ^b^	0.88 ± 0.11 ^b^
DBFE	1.12 ± 0.15 ^c^	1.20 ± 0.16 ^c^	1.18 ± 0.14 ^c^	1.15 ± 0.13 ^c^
DD	0.90 ± 0.13 ^b^	0.98 ± 0.12 ^b^	0.94 ± 0.11 ^b^	0.92 ± 0.10 ^b^
DHBFE	1.85 ± 0.19 ^c^	1.95 ± 0.20 ^c^	1.88 ± 0.18 ^c^	1.80 ± 0.17 ^c^

Histopathological scoring was performed using a blinded semi-quantitative scale ranging from 0 to 4, where 0 = normal histology and 4 = severe tissue damage. Values are expressed as means ± SEMs (n = 8). Different superscript letters within the same column indicate significant differences at *p* < 0.05 according to one-way ANOVA followed by Tukey’s HSD post hoc test.

**Table 11:** Binding affinities against target proteins associated with diabetes and thyroid regulation.

Compound	CID	Binding Affinity		
		Thyroid Hormone Receptor Beta (TRβ1) (PDB: 1NAX)	Peroxisome Proliferator-Activated Receptor Gamma (PPARγ) (PDB: 2PRG)	AMP-Activated Protein Kinase (AMPK) (PDB: 4CFF)
C1	7311	−7.8	−6.2	−6.7
C2	200981	−7.6	−6	−6.3
C3	21205	−7.3	−5.5	−5
C4	558370	−7.1	−6.2	−5.8
C5	985	−7.1	−5.3	−5.3
C6	151398	−6.9	−5.4	−5.9
C7	3016654	−6.7	−6.1	−5.6
C8	75802	−6.6	−5.6	−5.4
C9	518900	−6.5	−5.4	−5.5
C10	6269	−6.4	−5.6	−5.6
C11	12543919	−6.3	−5.8	−5.6
C12	31231	−6.3	−5.5	−5.6
C13	70133	−6.3	−5.8	−5.8
C14	5429	−6.2	−5.9	−5.7
C15	91712725	−6.1	−5.3	−5.2
C16	12570	−5.9	−5.6	−5.8
C17	892	−5.8	−5.7	−5.4
C18	2519	−5.8	−5.6	−5.7
C19	21987554	−5.5	−4.7	−4.9
C20	237332	−5.5	−4.9	−4.5
C21	69217	−5.4	−4.4	−4.8
C22	3726592	−5.3	−6.1	−6.2
C23	11233	−5.2	−4.4	−4.4
C24	5365371	−4.9	−5.9	−4.4
C25	31263	−4.9	−4.6	−4.4
C26	137721	−4.9	−4.5	−4.5
C27	538312	−4.9	−5.1	−5.1
C28	8201	−4.6	−4.8	−5.3
C29	1639	−4.4	−4.5	−4.8
C30	538803	−4.4	−4.3	−4.1
C31	16541	−4.2	−5.3	−5
C32	44983	−4.2	−3.7	−3.6
C33	7288	−4.1	−3.8	−3.5

**Table 12:** (**a**) Ligand interaction with amino acid residues of Thyroid Hormone Receptor Beta (TRβ1) (PDB: 1NAX) and their bond distances. (**b**) Ligand interaction with amino acid residues of Peroxisome Proliferator-Activated Receptor Gamma (PPARγ) (PDB: 2PRG) and their bond distances. (**c**) Ligand interaction with amino acid residues of AMP-Activated Protein Kinase (AMPK) (PDB: 4CFF) and their bond distances.

(**a**)					
**Ligand Serial**	**PubChem CID**	**Docking Score (kcal/mol)**	**Amino Acid (Distances: X)**	**Types**	**Category**
C1	7311	−7.8	ILE276 (2.86271)	Pi-Sigma	Hydrophobic
			LEU330 (4.52222), ILE276 (4.92644), LEU346 (4.76793)	Alkyl	
			PHE272 (5.27441), LEU330 (4.86611)	Pi-Alkyl	
C2	200981	−7.6	PHE272 (2.58851)	Conventional Hydrogen Bond	Hydrogen Bond
			ILE276 (2.93313)	Pi-Sigma	Hydrophobic
			PHE272 (4.93264)	Pi-Pi Stacked	
			ALA279 (4.20143), ILE275 (4.44541), ILE276 (4.49315), LEU330 (4.54324)	Alkyl	
			LEU330 (4.85113), LEU346 (4.80733)	Pi-Alkyl	
(**b**)					
Ligand Serial	PubChem CID	Docking Score (kcal/mol)	Amino Acid (Distances: X)	Types	Category
C4	558370	−6.2	GLN286 (2.95706)	Carbon Hydrogen Bond	Hydrogen Bond
			CYS285 (4.29324), ARG288 (5.0463), LEU330 (4.23518)	Alkyl	Hydrophobic
C1	7311	−6.2	LEU270 (2.5301)	Pi-Sigma	Hydrophobic
(**c**)					
Ligand Serial	PubChem CID	Docking Score (kcal/mol)	Amino Acid (Distances: X)	Types	Category
C1	7311	−6.7	VAL96 (2.14644)	Conventional Hydrogen Bond	Hydrogen Bond
			LEU22 (4.58441), LEU146 (4.77812), VAL30 (4.16148), LEU146 (5.28453)	Alkyl	Hydrophobic
			LEU22 (5.1324), VAL30 (5.32307), ALA43 (4.51427), LEU146 (4.3923)	Pi-Alkyl	
C2	200981	−6.3	VAL30 (2.41924),	Pi-Sigma	Hydrophobic
			ALA43 (3.70317), VAL96 (4.67098), LEU146 (5.10408)	Alkyl	
			TYR95 (4.67839), LEU22 (5.13237), VAL30 (4.64647), ALA43 (4.20424), LEU146 (4.87793), LYS45 (5.1789)	Pi-Alkyl	
			MET93 (4.96686)	Pi-Sulfur	Other

## Data Availability

The data used to support the results of the current study can be made accessible upon reasonable request to the corresponding author. The data cannot be made publicly available for privacy and/or ethical reasons.
